# Receptor-tethered orthogonal IL-2 enhances regulatory T cell therapy

**DOI:** 10.1084/jem.20251480

**Published:** 2026-08-12

**Authors:** Alyssa C. Indart, Huiyun Lyu, Vinh Q. Nguyen, Wendy Rosenthal, Jatin R. Palvai, Sophie S. Jang, Yue Chen, Kevin Jude, Leon Su, K. Christopher Garcia, Jeffrey A. Bluestone, Qizhi Tang

**Affiliations:** 1 Diabetes Center, University of California, San Francisco, San Francisco, CA, USA; 2 Gladstone-UCSF Institute of Genomic Immunology, San Francisco, CA, USA; 3 Department of Otolaryngology-Head and Neck Surgery, Stanford University, Stanford, CA, USA; 4 Department of Molecular and Cellular Physiology, Stanford University, Stanford, CA, USA; 5 Department of Surgery, University of California, San Francisco, San Francisco, CA, USA

## Abstract

Regulatory T cell (Treg) therapy is an emerging platform for controlling immune overactivation. Persistence of infused Tregs is limited by insufficient IL-2, which is essential for Treg survival and function. IL-2 activates many immune cells, imposing a challenge for the selective provision of IL-2 to infused Tregs. In this study, we found that infusions of orthogonal (ortho) IL-2 failed to enhance Tregs expressing a corresponding orthoIL-2 receptor (IL-2R) in a mouse model of autoimmune diabetes. Engineering Tregs with an orthoIL-2 tethered to its receptor achieved selective autocrine signaling; increased CD25, CTLA-4, and Foxp3 expression; supported Treg persistence without exogenous IL-2 *in vivo*; and improved the efficacy of Treg prevention of autoimmune diabetes. Inserting the tethered orthoIL-2 construct into the *Foxp3* locus enabled Treg-specific self-reinforced expression through the activation of the *Foxp3* locus by increased IL-2 signaling. Together, these results illustrate a safe and effective cell-engineering solution for overcoming Tregs’ dependency on exogenous IL-2, thereby achieving superior therapeutic efficacy.

## Teaser

Tregs engineered with an orthogonal IL-2 tethered to its receptor persisted without exogenous IL-2 and exhibited superior therapeutic efficacy in a mouse model of type 1 diabetes.

## Graphical Abstract

**Figure:**
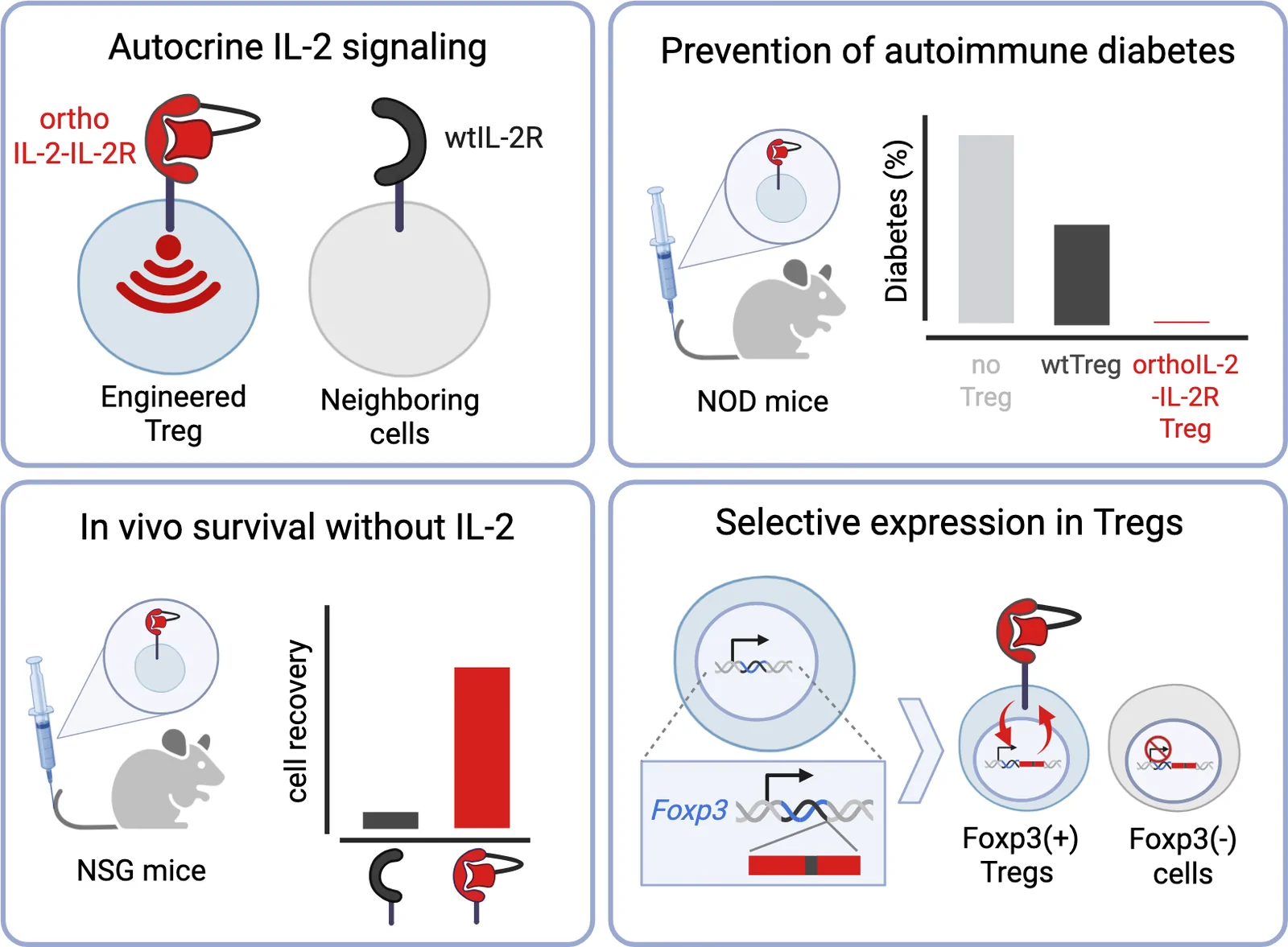

## Introduction

The essential function of regulatory T cells (Tregs) in maintaining immune homeostasis is illustrated by the life-threatening autoimmune syndrome in human and mice with mutations in the *FOXP3* gene, a Treg lineage–determining transcription factor (Dikiy and Rudensky, 2023; Hori, 2021; Ramsdell and Rudensky, 2020; Ramsdell and Ziegler, 2014; Sakaguchi et al., 2020). Moreover, the development of many more common autoimmune diseases can be attributed to an imbalance between Tregs and autoreactive T cells (Sumida et al., 2024). Treg therapy effectively suppresses a wide range of autoimmune and inflammatory diseases in preclinical models, prompting the ongoing research and development of Treg cell therapies to treat autoimmune, inflammatory, and degenerative diseases in humans (Bluestone et al., 2026).

Tregs constitutively express CD25, the interleukin 2 (IL-2) receptor (IL-2R) α chain, which stabilizes the trimeric IL-2, IL-2Rβ, and IL-2Rγ complex (Malek and Castro, 2010). This allows Tregs to respond to low concentrations of IL-2. IL-2, a T cell growth and survival factor produced by activated T cells (Chinen et al., 2016; Malek and Castro, 2010), plays an indispensable role in the development, proliferation, and survival of Tregs (Fontenot et al., 2005; Rubtsov et al., 2010; Setoguchi et al., 2005). IL-2 signaling reinforces Treg lineage identity by increasing Foxp3 expression and sustains high expression of CD25 and CTLA-4 that are important for Treg function (Fontenot et al., 2005; Jamison et al., 2024; Pandiyan et al., 2007). Paradoxically, Tregs do not produce IL-2 and are dependent on exogenous IL-2 produced by conventional T cells (Tconvs). Thus, the IL-2 and IL-2R axis plays a pivotal role in regulating the dynamics between immunosuppressive Tregs and immune-activating Tconvs (Busse et al., 2010; Feinerman et al., 2010; Fontenot et al., 2005; Jamison et al., 2024; Wong et al., 2021). Genome-wide association studies have revealed linkage of polymorphisms in *IL2* and *IL2RA* gene loci to various autoimmune diseases, including type 1 diabetes, multiple sclerosis, celiac disease, autoimmune hepatitis, and Crohn’s disease (Hartmann et al., 2014; Todd et al., 2007; van Heel et al., 2007), underscoring the importance of IL-2 signaling in immune self-tolerance.

**Figure 1.**
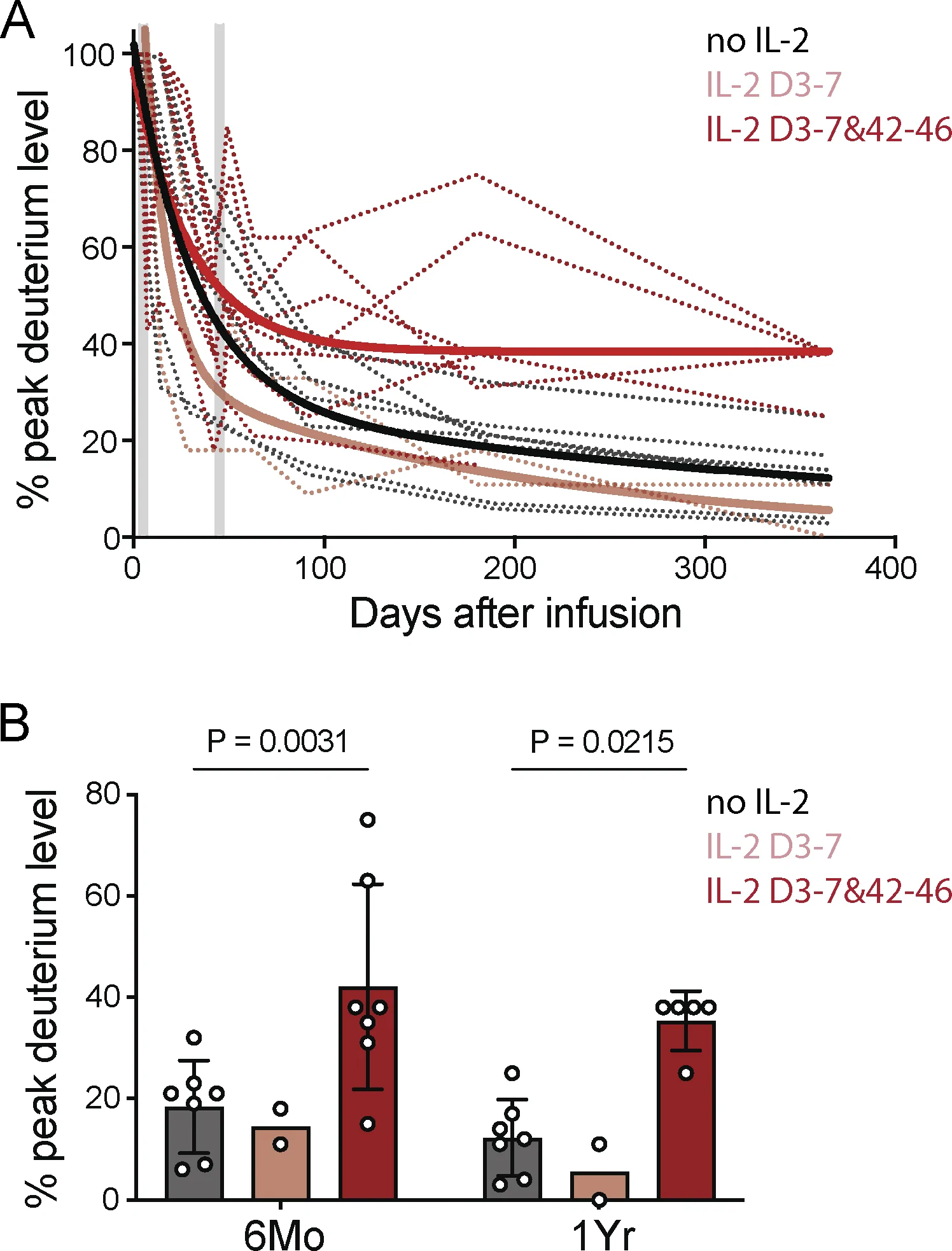
**Pharmacokinetics of infused Tregs in the peripheral blood of type 1 diabetes patients.** FACS-purified autologous Tregs were expanded *ex vivo* in medium containing deuterated glucose, which resulted in the enrichment of deuterium in the genome of the expanded Treg products. Percentage of deuterium enrichment in the peripheral blood Tregs can then be used to assess the pharmacokinetics of the Tregs after infusion. **(A)** Deuterium signals in peripheral blood over time. The black lines are results from patients who received Tregs alone. The dark red lines are results from patients who received Tregs followed by two rounds of daily IL-2 infusion given between days 3–7 and 42–46. The light pink lines are results from patients who received Tregs followed by one round of daily IL-2 infusion given between days 3–7. The thin dotted lines are data for individual patients, and the thick solid lines are the nonlinear two-phase decay fit of the group (R^2^ for no IL-2, 1× IL-2, and 2× IL-2 are 0.7721, 0.7209, and 0.4603, respectively). All data are normalized to peak deuterium enrichment detected between day 1 and 14 after cell infusion. **(B)** Normalized deuterium enrichment at 6 mo and 1 year after infusion in the three groups of patients were compared. Statistical significance was determined using mixed-effects analysis followed by Dunnett’s multiple comparisons test. Only statistically significant P values are listed. Results shown are a reanalysis of previously published data (Bluestone et al., 2015; Dong et al., 2021).

In a phase 1 Treg therapy trial in type 1 diabetes, we observed a loss of 75% of the infused Tregs within 3 mo of infusion (Bluestone et al., 2015). In a follow-up trial, we found co-infusion of low-dose IL-2 supported the persistence of the infused Tregs (Fig. 1) but also activated cytotoxic T cells (Dong et al., 2021). How to selectively enhance the survival and persistence of therapeutic Tregs is an important challenge to overcome. An orthogonal (ortho) IL-2 may achieve selective signaling in Tregs engineered with a corresponding orthoIL-2R (Sockolosky et al., 2018). In this study, we used the nonobese diabetes (NOD) mouse model of human type 1 diabetes to test this approach. Sustained stimulation of Tregs with low-dose IL-2 stalls autoimmune destruction of islet β cells in this model (Grinberg-Bleyer et al., 2010; Põder et al., 2026; Tang et al., 2008). Moreover, diabetes progression can be controlled by Tregs (Tang et al., 2004; Tarbell et al., 2004; Yang et al., 2022). We thus used this model to determine if an orthoIL-2–IL-2R system could synergize with therapeutic Tregs. We found that soluble orthoIL-2 was not sufficiently potent or selective to support therapeutic Tregs *in vivo*, which prompted us to develop a cell autonomous tethered orthoIL-2–IL-2R construct that sustained Treg survival and improved the potency of the Tregs in preventing autoimmune diabetes. Lastly, we show that targeting this orthoIL-2 autocrine construct to the Foxp3 locus improved the safety of the strategy by avoiding off-Treg expression and reinforced Treg lineage stability via a positive feedback loop.

## Results

### OrthoIL-2 3A10 was highly selective for orthoIL-2R^+^ Tregs but lacked potency *in vivo*

**Figure 2.**
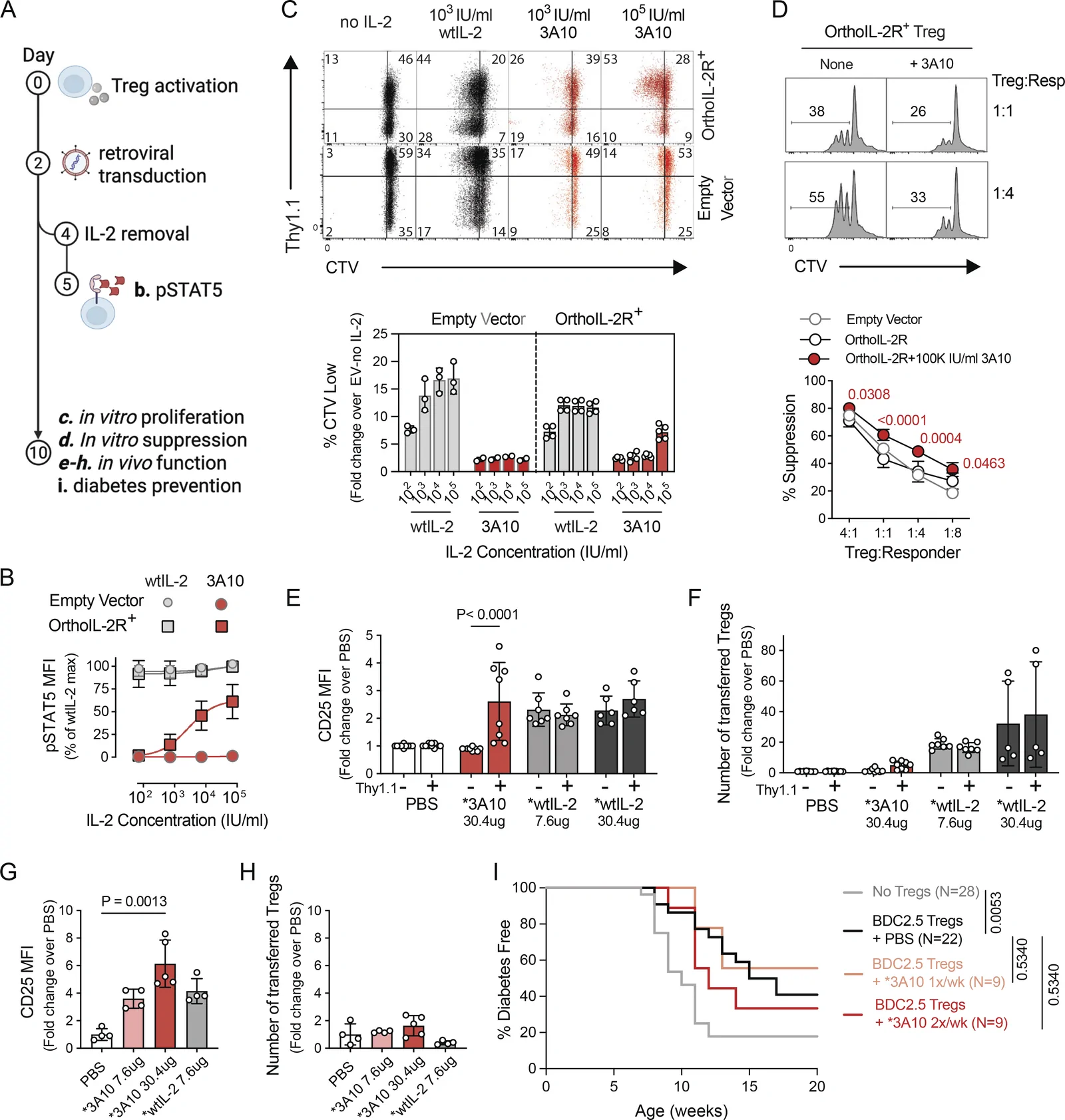
**OrthoIL-2 3A10 is a selective but weak agonist for orthoIL-2R. (A)** Experimental workflow for assessing function of 3A10 *in vitro* and *in vivo*. Created in BioRender. Tang (2026) https://BioRender.com/q07c632. **(B)** Dose response of IL-2–induced pSTAT5 in Tregs transduced with EV or orthoIL-2R with titrated concentrations of wtIL-2 or orthoIL-2 3A10. **(C)** Treg proliferation was measured using CTV dilution. **(D)***In vitro* suppression. Ordinary two-way ANOVA followed by Tukey’s multiple comparison after test was used to determine the statistical significance of the difference observed. P values on the graph are for comparisons between orthoIL-2R^+^ Tregs with or without added 100,000 IU/ml 3A10 at each Treg:responder ratio. Results in B–D are a summary of three independent experiments (means ± SD, *n* = 3). **(E and F)** NSG mice were injected with orthoIL-2R^+^ Tregs and followed by seven daily infusions of MSA-fusion proteins (MSA indicated by *) or PBS as shown. CD25 expression (E) and number of transferred Tregs (F) in the spleens normalized to the means of PBS control are summarized (means ± SD, *n* = 8–10 mice per group from three experiments). Two-way ANOVA followed by Sidak multiple comparison after test was used to assess statistical significance of the difference between orthoIL-2R^−^ and orthoIL-2R^+^ cells under each treatment condition. **(G and H)** NOD.CD28KO mice were injected with BDC2.5 orthoIL-2R^+^ Tregs and followed by seven daily infusions of MSA-fusion proteins or PBS as shown. CD25 MFI and number of transferred Tregs in the spleens are summarized (means ± SD, *n* = 4–5 mice per group pooled from three experiments). Kruskal–Wallis test was used to determine the statistical significance from the PBS-treated controls. **(I)** NOD.CD28KO mice were treated with either 2,000 BDC2.5 Treg + PBS or BDC2.5 orthoIL-2R^+^ Tregs + MSA-3A10 30 μg once or twice per week for 15 wk. Mice with two readings of blood glucose >250 mg/dl were considered diabetic (*n* = 9–28 mice per group pooled from two experiments). Statistical significance was determined using Kaplan–Meier survival analysis. P values were calculated using Mantel–Cox test comparing each condition with the BDC2.5 Treg-treated group. For D, E, G, and H, only statistically significant P values are listed.

A mouse orthoIL-2 system has been developed by screening for ligands that preferentially bind to a mutated IL-2Rβ. Among a panel of orthoIL-2s generated, 3A10 is highly selective for orthoIL-2R with no measurable activity toward wtIL-2Rβ (Sockolosky et al., 2018). We thus expressed the orthoIL-2Rβ in NOD Tregs via retroviral transduction and tested effect of 3A10 *in vitro* and *in vivo* (Fig. 2 A). NOD Tregs transduced with the orthoIL-2R, but not the empty vector (EV), responded to 3A10 by phosphorylating STAT5 (pSTAT5) with an EC50 of 8,620 IU/ml (Fig. 2 B). In contrast, wtIL-2–induced maximal pSTAT5 in both EV- and orthoIL-2R-transduced Tregs at the lowest concentration tested (100 IU/ml, Fig. 2 B). Furthermore, 100,000 IU/ml 3A10–induced proliferation of orthoIL-2R^+^, but not EV-transduced, Tregs (Fig. 2 C), and mildly enhanced the function of orthoIL-2R^+^ Tregs in an *in vitro* suppression assay (Fig. 2 D).

**Figure S1.**
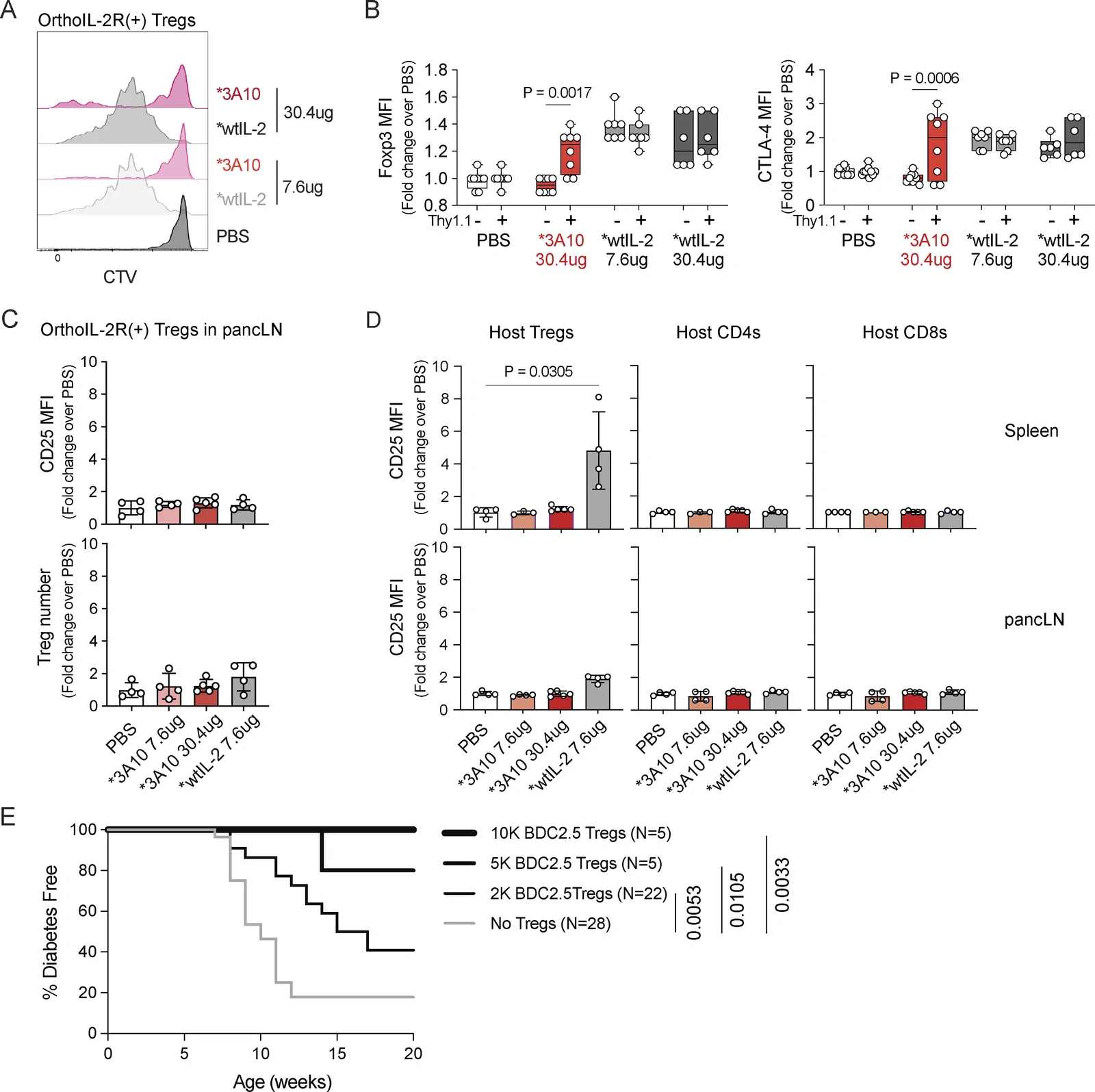
**OrthoIL-2 3A10 on orthoIL-2R engineered Tregs (related to Fig. 2). (A and B)** A mixture of orthoIL-2R–transduced Thy1.1^+^ and non-transduced Thy1.1^−^ NOD Tregs were transferred to NSG mice followed by daily i.p. injection of PBS, MSA-3A10, or MSA-wtIL-2 (MSA is denoted by *). Spleens were collected on day 3 after Treg transfer for assessing CTV dilution (A) and on day 8 for measuring Foxp3 and CTLA-4 expression (B). Ordinary two-way ANOVA followed by Sidak multiple comparison test was used to determine the statistical significance. **(C and D)** FACS-purified orthoIL-2(+) BDC2.5 Tregs were transferred to 5-wk-old NOD.CD28KO mice, followed by 7 daily i.p. injection of PBS, MSA-3A10, or MSA-wtIL-2. **(C)** CD25 expression on orthoIL-2(+) and total number of orthoIL-2R(+) Tregs in the pancLN. **(D)** CD25 expression on host T cells in the spleen and pancLN. Results shown are summaries of three to five independent experiments. Kruskal–Wallis test was used to determine the statistical significance. **(E)** A titrated doses of BDC2.5 Tregs were injected into 5-wk-old NOD.CD28KO mice to assess effect on diabetes incidence. Mantel–Cox log-rank test was used to determine the statistical significance. Only statistically significant P values are listed.

To test the efficacy of 3A10 on orthoIL-2R^+^ Tregs *in vivo*, we transferred orthoIL-2R^+^ Tregs along with non-transduced Tregs into immunodeficient NOD-scid IL-2Rgamma^null^ (NSG) mice that lack T cell–derived IL-2. The recipient mice received daily infusions of mouse serum albumin (MSA)-3A10 fusion protein, MSA-wtIL-2, or PBS as a control. The MSA fusion proteins were used to extend the half-life of 3A10 and wtIL-2 from 5 to 50 h (Sockolosky et al., 2018). High-dose 3A10 (30.4 μg daily) induced weak proliferation (Fig. S1 A) and selectively increased CD25, Foxp3, and CTLA-4 expression on Thy1.1^+^ orthoIL-2R^+^ Tregs (Fig. 2 E and Fig. S1 B). In addition, MSA-3A10 mildly increased the number of Thy1.1^+^ orthoIL-2R^+^ but not Thy1.1^−^ Tregs (Fig. 2 F). These data showed that orthoIL-2 3A10 was highly selective for orthoIL-2R^+^ Tregs but only weakly stimulated orthoIL-2R^+^ Tregs *in vitro* and *in vivo*.

To determine the impact of MSA-3A10 on the function of orthoIL-2R^+^ Tregs in lympho-replete recipients, we investigated the homeostasis and function of orthoIL-2R^+^ in NOD.CD28KO mice. These mice develop diabetes at an accelerated tempo and higher penetrance due to defective Treg development and peripheral homeostasis, thus providing a useful *in vivo* model for evaluating Treg function in autoimmune diabetes (Mahne et al., 2015; Obarorakpor et al., 2023; Salomon et al., 2000; Tang et al., 2004). In these experiments, we used islet antigen-specific Tregs from BDC2.5 TCR transgenic mice (referred to as BDC2.5 Tregs hereafter) because of their established efficacy in this model. The effects of MSA-3A10 were muted in NOD.CD28KO mice when compared with those observed in NSG mice. Despite the significant increase in CD25 expression on transferred orthoIL-2R^+^ Tregs, their total cell numbers were not different from PBS-treated controls in the spleen (Fig. 2, G and H). In the pancreatic LN (pancLN) where the cognate antigen for the BDC2.5 TCR was present, no increase in either CD25 expression or the number of orthoIL-2R^+^ Tregs was seen (Fig. S1 C). The mild effect of MSA-3A10 in the spleen was selective for orthoIL-2R^+^ Tregs since no change in CD25 expression was observed in the spleen or pancLN on endogenous Tregs, CD4^+^, and CD8^+^ Tconvs (Fig. S1 D). These results show that MSA-3A10 had limited ability to promote proliferation of orthoIL-2R^+^ Tregs in NOD.CD28KO mice.

We next determined if MSA-3A10 could enhance the efficacy of Tregs in preventing autoimmune diabetes. Previously, we have shown that 50,000–150,000 BDC2.5 Tregs prevented diabetes in 100% of NOD.CD28KO recipients (Spence et al., 2018; Tang et al., 2004). A dose titration experiment showed that 10,000 BDC2.5 Tregs were similarly effective, whereas 2,000 BDC2.5 Tregs were partially protective (Fig. S1 E). We thus infused mice with 2,000 BDC2.5^+^ orthoIL-2R^+^ Tregs and assessed the impact of MSA-3A10 supplements. No improvement in diabetes-free survival was observed (Fig. 2 I). Thus, despite being a highly selective ligand for the orthoIL-2R, 3A10 had limited *in vivo* efficacy in potentiating orthoIL-2R^+^ Tregs.

### OrthoIL-2 1G12 was more potent but lacked selectivity for orthoIL-2R^+^ Tregs

**Figure 3.**
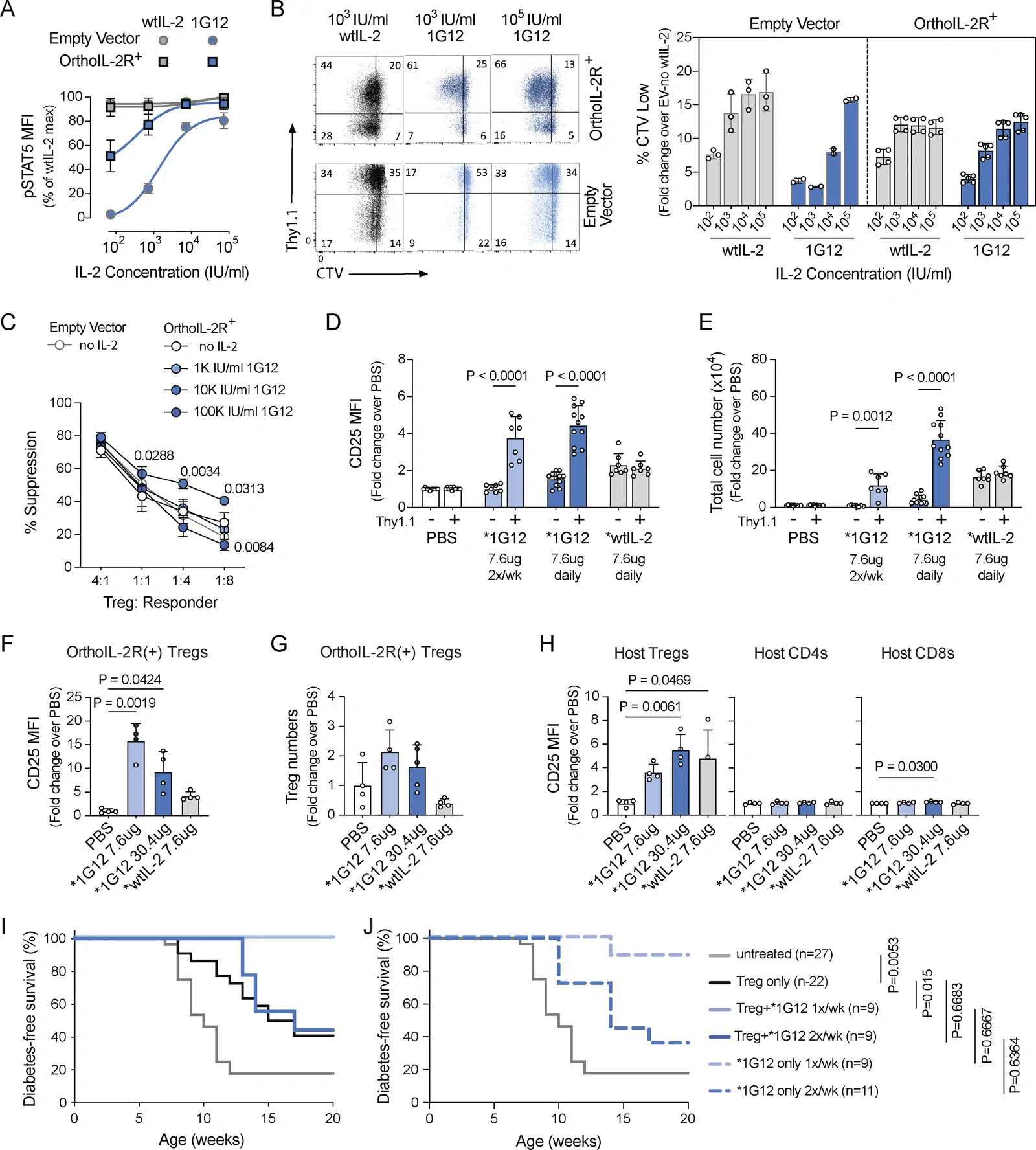
**OrthoIL-2 1G12 has higher potency but cross-reacts on wtIL-2R. (A)** Dose response of IL-2–induced pSTAT5 in Tregs transduced with EV or orthoIL-2R. **(B)** Treg proliferation measured using CTV dilution. The data were collected in the same experiment as shown in Fig. 2 C, thus the wtIL-2 control data are the same. **(C)***In vitro* suppression assay. Results shown in A–C are summaries of three independent experiments (means ± SD, *n* = 3). Ordinary two-way ANOVA followed by Tukey’s multiple comparison after test was used to determine the statistical significance of the difference observed. P values on the graph are for comparisons with orthoIL-2R^+^ Tregs without addition of 1G12 at each Treg:responder ratio. **(D and E)** NSG mice were injected with a mixture of orthoIL-2R–transduced Thy1.1^+^ and non-transduced Thy1.1^−^ NOD Tregs followed by treatment with PBS or MSA-fusion proteins (MSA indicated by *) at the doses shown on the graph. CD25 expression (D) and number of transferred Tregs (E) in the spleens are summarized. Numbers shown are normalized to the means of PBS control are summarized (means ± SD, *n* = 8–10 mice per group from three experiments). Two-way ANOVA followed by Sidak multiple comparison after test was used to assess statistical significance of the difference between orthoIL-2R^−^ and orthoIL-2R^+^ cells under each treatment condition. **(F–H)** NOD.CD28KO mice were injected with orthoIL-2R^+^ BDC2.5 Tregs and followed by seven daily injection of PBS or MSA-fusion proteins at the doses indicated. CD25 expression on transferred Tregs (F), number of transferred Tregs (G), and CD25 MFI on host T cells (H) in the spleens are summarized (means ± SD, *n* = 4–5 mice per group from three experiments). Kruskal–Wallis test was used to determine the statistical significance from the PBS-treated controls. **(I and J)** NOD.CD28KO mice were treated with PBS or 7.6 μg MSA-1G12 from 5 to 20 wk of age with (I) or without (J) 2,000 orthoIL-2R^+^ BDC2.5 Tregs. Mice with two readings of blood glucose >250 mg/dl were considered diabetic (*n* = 9–28 mice per group pooled from two experiments). Statistical significance was determined using Kaplan–Meier survival analysis. P values were calculated using Mantel–Cox test comparing each condition to BDC2.5 Treg-treated group and comparing the same dose of MSA-1G12 with or without BDC2.5 Tregs. For C–H, only statistically significant P values are listed.

**Figure S2.**
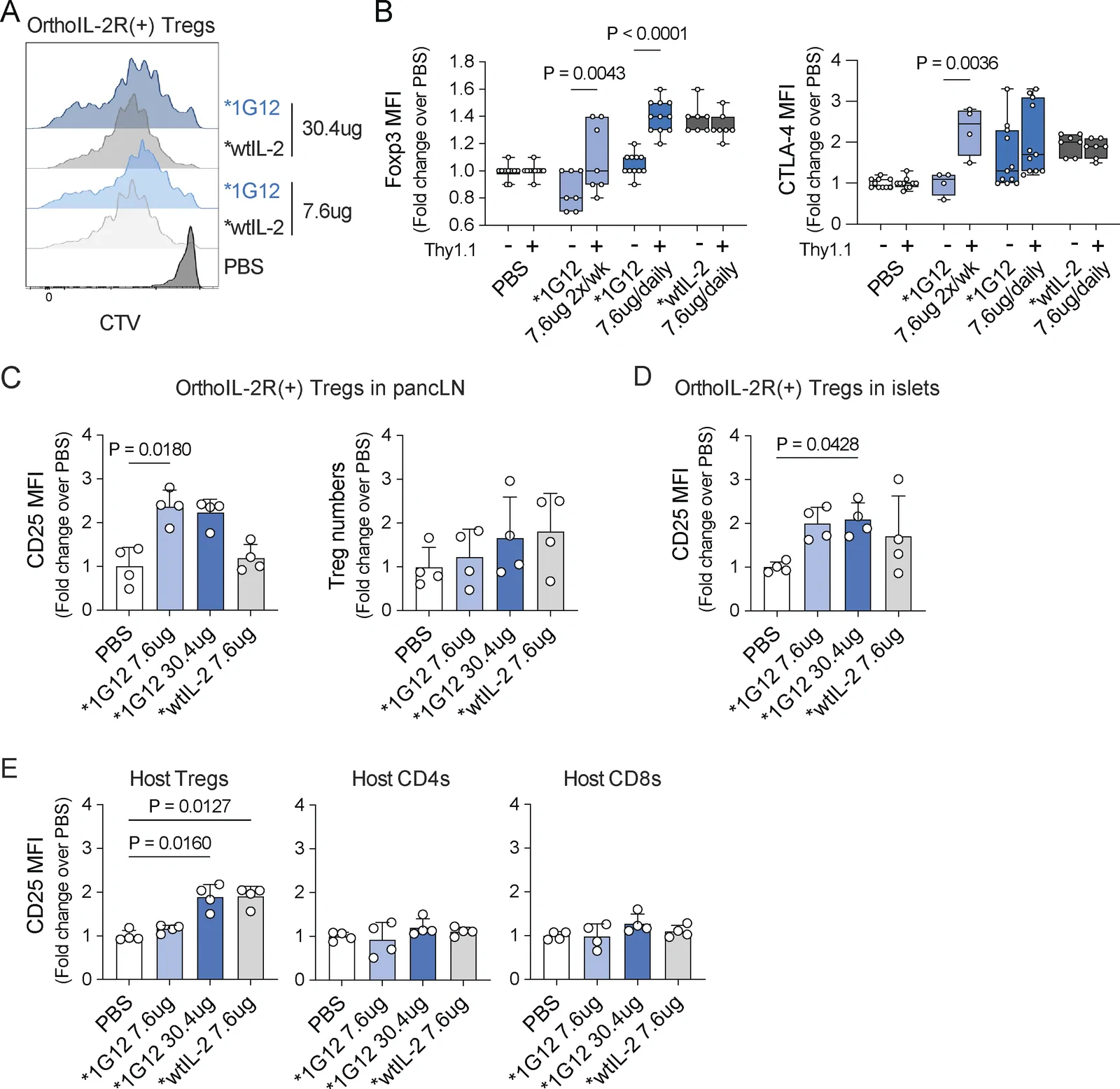
**OrthoIL-2 1G12 on orthoIL-2R engineered Tregs (related to Fig. 3). (A and B)** A mixture of orthoIL-2R–transduced Thy1.1^+^ and non-transduced Thy1.1^−^ NOD Tregs were transferred to NSG mice (200,000/mouse). **(A)** Mice received daily i.p. injection of PBS, MSA-1G12, or MSA-wtIL-2 at dose indicated (MSA is denoted by *). Spleens were collected on day 3 after transfer for assessing CTV dilution. **(B)** Mice were treated with MSA-1G12 or MSA-wtIL-2 at the dose indicated. Spleens were collected on day 8 to assess Foxp3 and CTLA-4 expression. Ordinary two-way ANOVA followed by Sidak multiple comparison test was used to determine the statistical significance. **(C–E)** FACS-purified orthoIL-2(+) BDC2.5 Tregs were transferred to 5-wk-old prediabetic NOD.CD28KO mice followed by seven daily i.p. injections of PBS, MSA-3A10, or MSA-wtIL-2 at the dose indicated on the graph. **(C)** CD25 expression on orthoIL-2(+) and total number of orthoIL-2R(+) Tregs in the pancLN. **(D)** CD25 expression on orthoIL-2(+) cell in the islets. **(E)** CD25 expression on host T cells in pancLN. Kruskal–Wallis test was used to determine the statistical significance of the difference with PBS controls. Only statistically significant P values are listed.

Realizing the limitation of 3A10’s low affinity, we explored the performance of another orthoIL-2, 1G12, that had higher affinity for orthoIL-2R^+^ but could also signal via wtIL-2R at high concentrations (Sockolosky et al., 2018). 1G12 was more potent than 3A10 with pSTAT5 EC_50_ of 100 IU/ml in orthoIL-2R^+^ Tregs. It also showed agonist activity in EV-transduced wtIL-2R–expressing Tregs with pSTAT5 EC_50_ of 1,714 IU/ml (Fig. 3 A and Fig. S2 A). 1G12 induced *in vitro* proliferation of orthoIL-2R^+^ Tregs but not in EV-transduced Tregs at low concentrations (1,000 IU/ml) (Fig. 3 B). At concentrations above 10,000 IU/ml, 1G12 induced proliferation of both orthoIL-2R^+^ and EV-transduced Tregs (Fig. 3 B). Similarly, 1G12’s effect on *in vitro* suppression was dose dependent. At 1,000 IU/ml, no change in the suppressive activity of orthoIL-2R^+^ Tregs was observed; slightly enhanced suppression was seen at 10,000 IU/ml of 1G12, whereas significantly impaired suppression was seen with 100,000 IU/ml of 1G12 at 1:8 Treg:Tconv ratio (Fig. 3 C), likely due to its activation of responder T cells in the assay.

In NSG mice, orthoIL-2R^+^ Tregs proliferated comparably when the mice received daily infusion of 7.6 or 30.4 μg of MSA-1G12 or MSA-wtIL-2 (Fig. S2 A), suggesting that daily infusion of 7.6 μg of either IL-2 form was saturating. We thus eliminated the 30.4 μg dose in follow-up experiments and added 7.6 μg twice per week dosing. We observed that daily and twice per week MSA-1G12 were similarly effective in inducing CD25, Foxp3, and CTLA-4 expression on orthoIL-2R^+^ Tregs (Fig. 3 D and Fig. S2 B), but daily dosing induced a significantly greater increase in the number of orthoIL-2R^+^ Tregs when compared with twice per week dosing (Fig. 3 E). Moreover, neither dose of MSA-1G12 significantly changed CD25 expression and the cell numbers of Thy1.1^−^ orthoIL-2R^−^ cells in these short-term experiments (Fig. 3, D and E).

In lympho-replete NOD.CD28KO recipients, 7.6 and 30.4 μg daily MSA-1G12 significantly increased the expression of CD25 on transferred orthoIL-2R^+^ Tregs in the spleen (Fig. 3 F), pancLN (Fig. S2 C), and pancreatic islets (Fig. S2 D). However, the total numbers of infused orthoIL-2R^+^ Tregs in the spleens (Fig. 3 G) and pancLNs (Fig. S2 C) were not significantly more than PBS-treated controls. Daily infusion of 30.4 μg MSA-1G12 did not change the numbers of host CD4^+^ or CD8^+^ Tconvs in the spleens and pancLN (Fig. S2 E), but significantly increased CD25 expression on host Treg in the spleen (Fig. 3 H) and pancLN (Fig. S2 E), indicating cross reactivity on host cells at this high dose.

We then treated NOD.CD28KO mice with 2,000 BDC2.5 orthoIL-2R^+^ Tregs followed by 7.6 μg of MSA-1G12 once or twice a week. While mice that received once per week MSA-1G12 were all protected from diabetes, those that received twice a week MSA-1G12 developed diabetes at the same rate as Treg alone (Fig. 3 I). Since MSA-1G12 could act on endogenous Tregs and Tconvs, we treated NOD.CD28KO mice once or twice a week with 7.6 μg MSA-1G12 without infusion of Tregs. Diabetes incidences mirrored those with Treg infusion (Fig. 3 J). Thus, 1G12 controlled diabetes independent of the infused Tregs, and its effect was dose dependent, similar to previous observations with wtIL-2 (Grinberg-Bleyer et al., 2010; Tang et al., 2008). These data together show the limited utility of 1G12 as an adjunct therapy to therapeutic Tregs.

### Potency and selectivity of tethered orthoIL-2–IL-2Rβ system depended on the ligand and the linker

**Figure 4.**
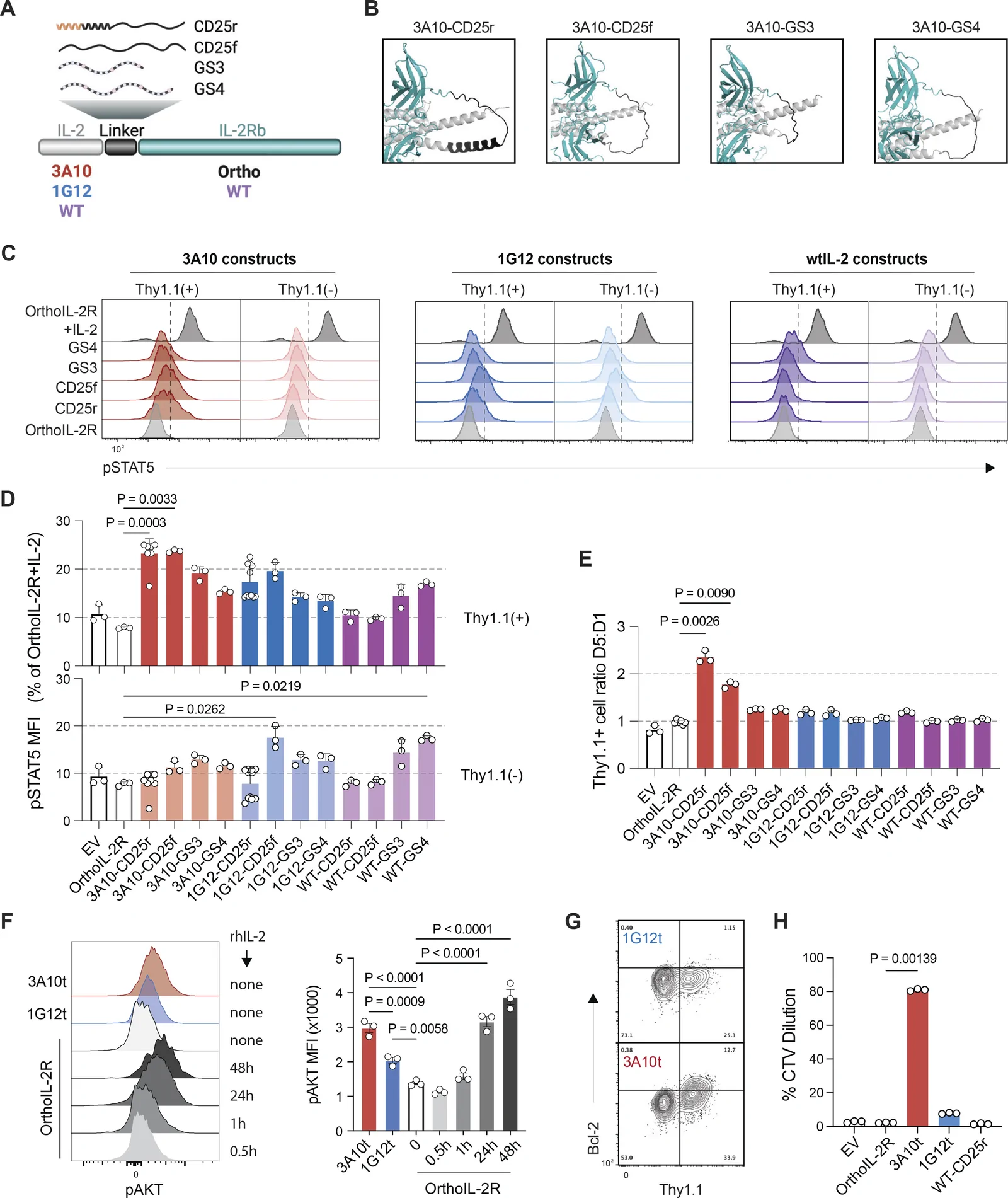
**Engineering a tethered orthoIL-2–IL-2Rβ system. (A)** Schematic of the tethered IL-2–IL-2R components tested. Created in BioRender. Tang (2026) https://BioRender.com/dayo897. **(B)** Visualization of the four linkers used to tether orthoIL-2 3A10 to orthoIL-2R using PyMOL. **(C and D)** Flow cytometric plots of pSTAT5 in transduced Tregs (Thy1.1^+^, left) or not (Thy1.1^−^, right) with various constructs indicated. Representative flow plots are shown. **(C)** Results were repeated in three independents experiments. **(D)** Data were expressed as percentages of pSTAT5 MFI relative to that induced in orthoIL-2R–transduced Tregs stimulated with 100 IU/ml mouse IL-2. **(E)** Enrichment of Thy1.1^+^ Tregs on day 5 versus day 1 after exogenous IL-2 withdrawal. Results shown are a summary of three independent experiments (mean ± SD, *n* = 3–6). **(F)** pAKT in 3A10-CD25r (3A10t), 1G12-CD25r (1G12t), and orthoIL-2R engineered Tregs. Aliquots of orthoIL-2R Tregs were stimulated with 2,000 IU/ml rhIL-2 for the indicated duration as a reference. **(G)** Representative flow cytometric plots of Bcl-2 expression in Tregs transduced with 3A10t and 1G12t constructs. The results shown are representative of three independent experiments. **(H)** On day 5 after activation, Thy1.1^+^ Tregs were purified, labelled with CTV, and cultured without TCR activation or exogenous IL-2. CTV dilution was measured on day 14. For D–F and H, Kruskal–Wallis test was used to determine the statistical significance when compared with the orthoIL-2R controls. For all panels, only statistically significant P values are shown.

**Figure S3.**
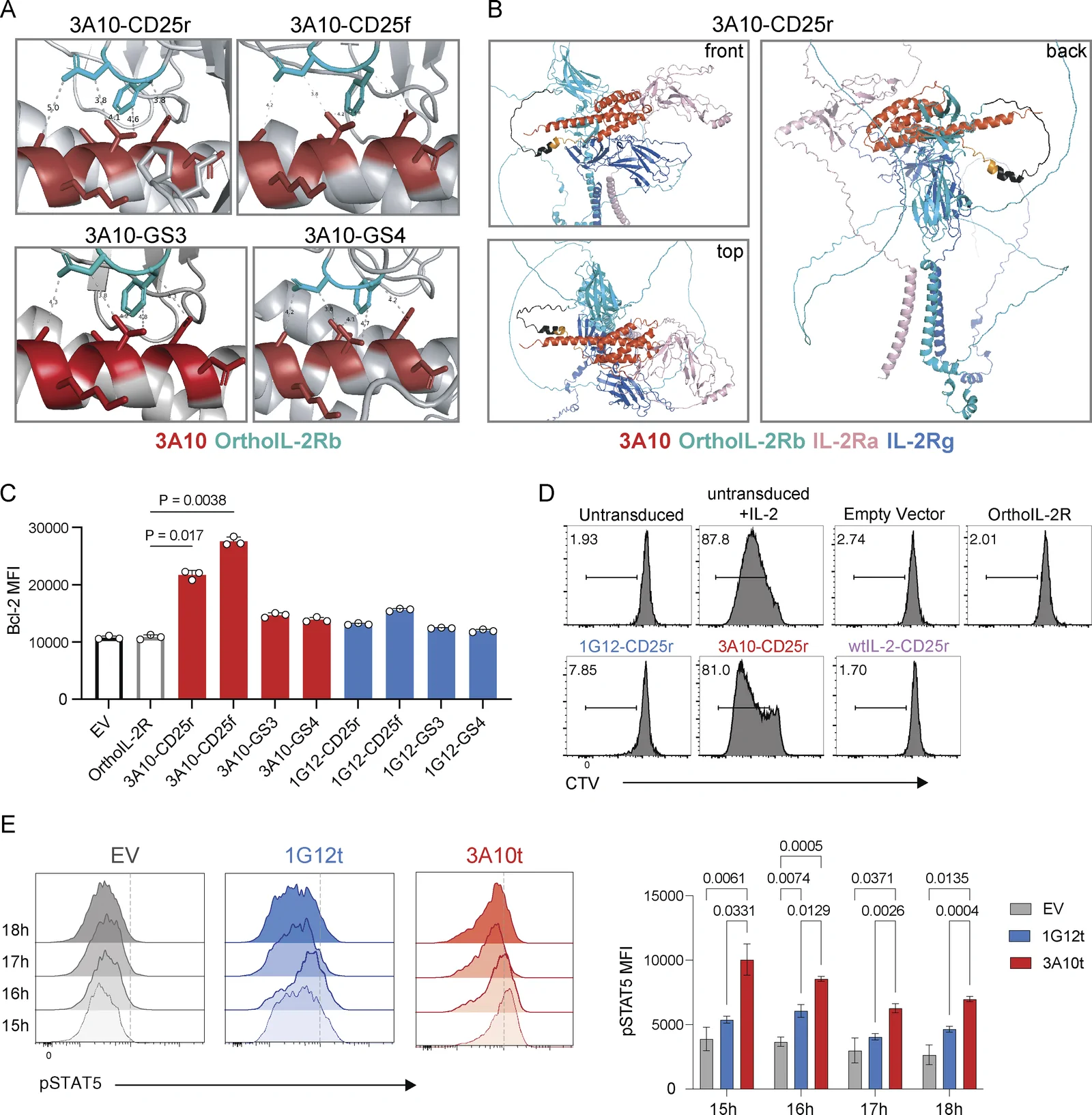
**Structure prediction and function assessment of orthoIL-2–IL-2R constructs (related to Figs. 4 and 5). (A and B)** Alpha-fold models showing the distance of 3A10’s side chains from the orthoIL-2Rβ–binding pocket when tethered to all four linkers (A) and 3A10-CD25r and the trimeric IL-2R complex (B). **(C and D)** Functional assessment of various tethered constructs. **(C)** Expression of Bcl-2 in Tregs 5 days after withdrawal of exogenous IL-2 (related to Fig 4 G). **(D)** CTV dilution profile of Fig 4 H. **(E)** Tregs were removed from exogenous IL-2 immediately after spinfection on day 2 after activation. The cells were cultured in the absence of exogenous IL-2 for 15–18 h before flow cytometric analysis of pSTAT5. Representative flow cytometry profiles of pSTAT5 are shown on the left and pSTAT5 MFI are summarized in the graph on the right. Statistical significance of the difference was determined using two-way ANOVA followed by Tukey’s multiple comparison test.

We hypothesized that physically tethering the orthoIL-2 to the orthoIL-2R would improve the potency and selectivity of the orthoIL-2–IL-2R system. We thus designed four linkers containing 15–26 amino acids, which should be sufficient to span the distance of 41 Å between the C terminus of the IL-2 and the N terminus of the IL-2Rβ (Fig. 4 A). The first linker was a 26-amino acid peptide from the extracellular domain of CD25 (Jounaidi et al., 2017). The linker contains a short rigid α helix followed by a flexible disordered domain of CD25, which we named CD25-rigid (CD25r). The second linker was a 26-amino acid peptide entirely from the disordered region of the CD25 extracellular domain to give the linker more flexibility. We termed this linker CD25-flexible (CD25f). The other two linkers were three or four repeats of GGGGS, named GS3 and GS4, respectively. We used each of these linkers to tether 3A10 or 1G12 to orthoIL-2Rβ. We also generated four analogous wtIL-2 tethered to wtIL-2Rβ with these linkers to test if tethering IL-2 would be sufficient to restrict IL-2 signaling to the engineered cells to obviate the need for orthogonality. AlphaFold models showed that the side chains of tethered 3A10 were 3–6 Å away from the orthoIL-2Rβ–binding pocket (Fig. 4 B and Fig. S3 A) and there was no steric hindrance from the linkers (Fig. S3 B).

Tregs expressing the 3A10 with CD25r and CD25f linkers had significantly higher pSTAT5 when compared with Tregs transduced with orthoIL-2Rβ alone and the 3A10 constructs with the two GS linkers showed milder increases. Moreover, Thy1.1^−^ non-transduced Tregs in the same culture of all 3A10 constructs showed minimal increase of pSTAT5 (Fig. 4, C and D). Tregs expressing all tethered 1G12 constructs had increased pSTAT5 in Thy1.1^+^ and some showed increased pSTAT5 in Thy1.1^−^ Tregs (Fig. 4, C and D). Lastly, wtIL-2-GS3 and GS4, but not the CD25-based linkers, showed increased pSTAT5 in Thy1.1^+^ and Thy1.1^−^ Tregs (Fig. 4, C and D). Thus, despite physically linking the IL-2 to its receptor, the tethered 1G12 and wtIL-2 could signal in neighboring non-engineered wtIL-2R–expressing cells depending on the linker used. Tregs engineered with 3A10-CD25r and 3A10-CD25f were significantly enriched between days 1 and 5 after IL-2 withdrawal (Fig. 4 E). In addition to activation of STAT5, IL-2 signaling also activates PI3K–mTOR pathway that regulate Treg metabolism and survival. We thus examined phosphorylated AKT (pAKT), downstream of PI3K. Tregs engineered with orthoIL-2R without a tethered ligand responded to exogenous IL-2 by increasing pAKT over a 48-h period. Both 3A10-CD25r (renamed 3A10t)- and 1G12-CD25r (renamed 1G12t)-engineered Tregs showed significantly increased pAKT in the absence of exogenous IL-2, and the signal was significantly higher in 3A10t Tregs when compared with 1G12t Tregs (Fig. 4 F). Consistent with these signaling differences, 3A10t Tregs showed increased Bcl-2 expression (Fig. 4 G and Fig. S3 C) and more active proliferation (Fig. 4 H and Fig. S3 D). These *in vitro* experiments identified 3A10 tethered to orthoIL-2R with the CD25 linkers as promising candidates for selective and cell autonomous support of Treg survival and proliferation.

### Transcriptomic program of Tregs expressing tethered orthoIL-2–IL-2R

**Figure 5.**
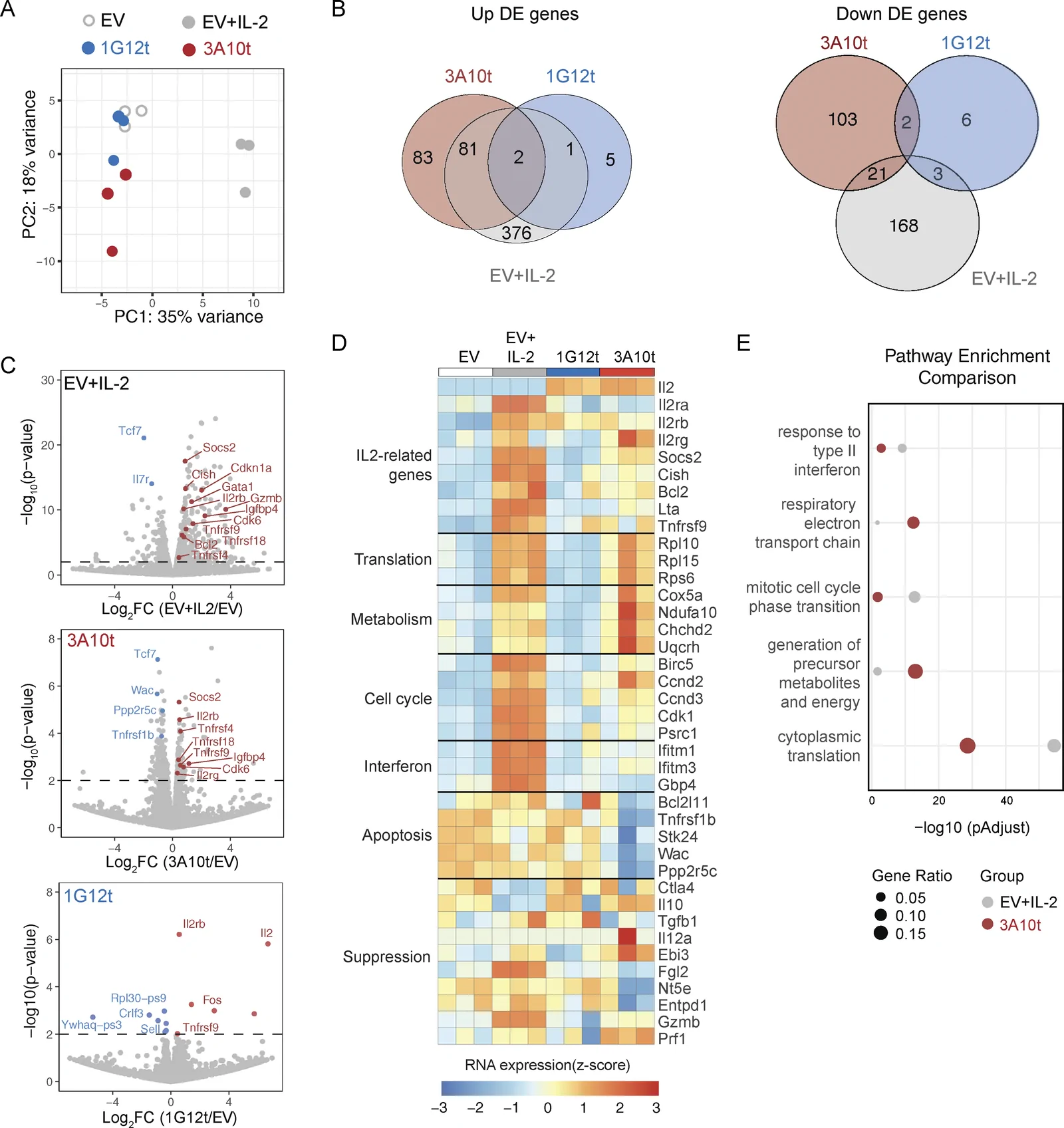
**RNA-seq analysis of engineered Tregs. (A)** PC analysis of RNA-seq data of Treg cells expressing EV with or without IL-2, and Tregs expressing 1G12t or 3A10t. **(B)** Venn diagrams showing overlap of upregulated (left) and downregulated (right) genes in EV+IL-2, 1G12t and 3A10t Tregs when compared with EV without IL-2 samples. **(C)** Volcano plots of DEGs in EV+IL-2 (top), 3A10t (middle), and 1G12t (bottom) Tregs when compared with EV. Horizontal dashed lines indicate P value of 0.01. Please refer to Data S1 for a complete list of DE genes. **(D)** Heatmap displaying expression of selected genes in EV, EV+IL-2, 1G12t, and 3A10t Tregs, grouped by pathways. **(E)** Gene Ontology (GO) pathway analysis showing the top five featured pathways among genes significantly upregulated in EV+IL-2 and 3A10t Tregs. The plot depicts the adjusted P values of DEGs in each pathway with circle size indicating the gene ratio of DEGs within the pathway. Source data are available for this figure: SourceData F5.

The finding that the weak agonist, 3A10, induced stronger IL-2 signaling when tethered to the IL-2R was unexpected. 1G12 has higher affinity for orthoIL-2R than 3A10; it is possible that the tethered 1G12 was more efficient in inducing orthoIL-2R endocytosis and degradation (Cendrowski et al., 2016; Chen et al., 2017). It is also possible that 1G12t induced strong negative feedback (e.g., *Socs* and *Cish* expression) (Alexander and Hilton, 2004; Sobah et al., 2021), resulting in the termination of signaling. Lastly, persistent signaling by 1G12t might have led to epigenetic silencing or cell death (Moro et al., 2022). We performed bulk RNA sequencing (RNA-seq) of 3A10t- and 1G12t-transduced Tregs in the absence of IL-2 to explore these possibilities. EV-transduced Tregs with or without 100 IU/ml mouse IL-2 were included as controls. Principal component (PC) analysis revealed EV Tregs with IL-2 were most distinct and separated from others along the PC 1 axis. The remaining samples separated along the PC2 axis with 1G12t and EV no IL-2 Tregs clustered closer together (Fig. 5 A).

**Figure S4.**
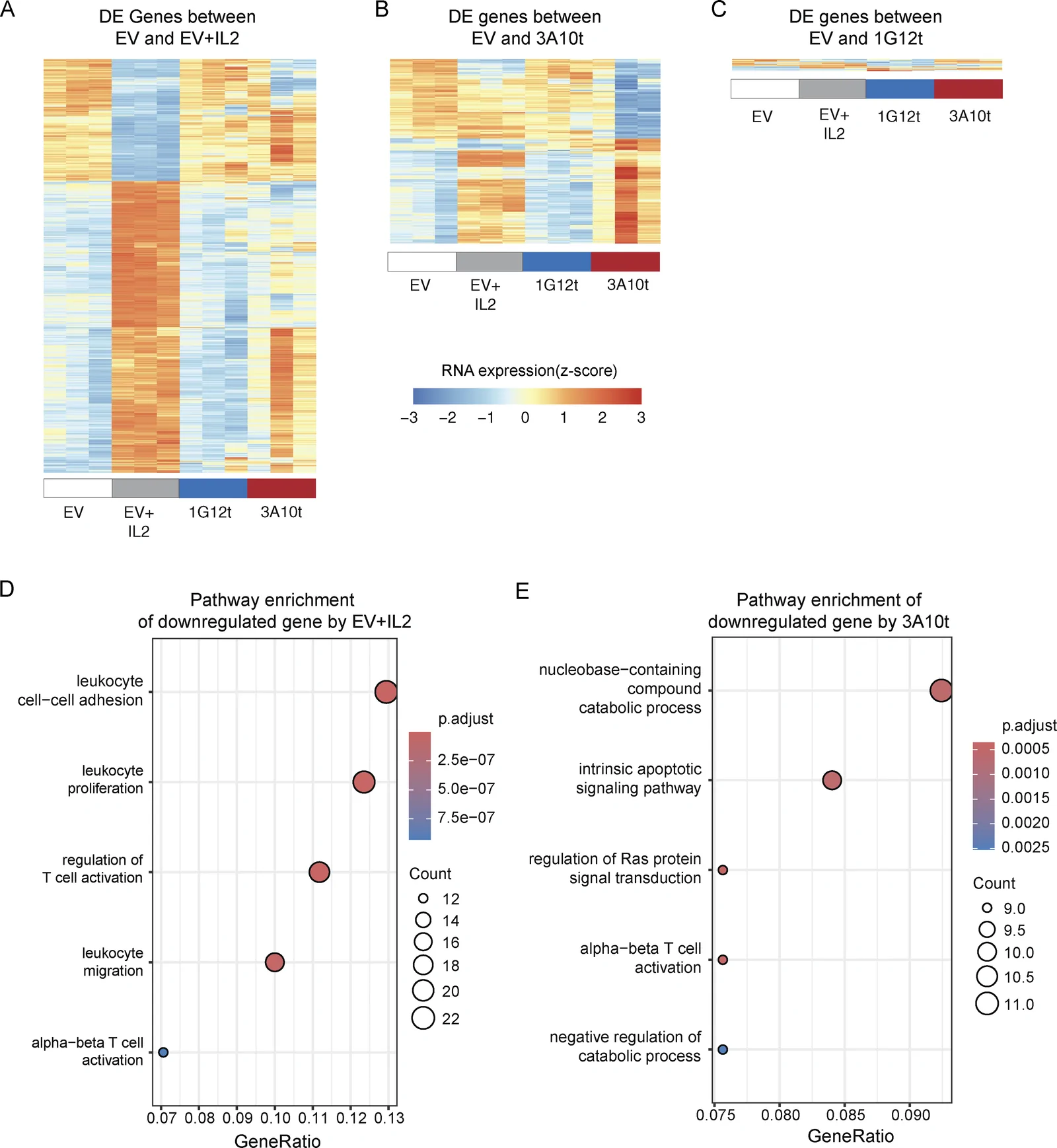
**Distinct transcriptomic program in Tregs expressing various tethered orthoIL-2 constructs (related to Fig. 5). (A)** Heatmap of the expression of DE genes identified between EV+IL-2 and EV by all samples are shown. **(B)** Heatmap of the expression of DE genes identified between 3A10t and EV by all samples are shown. **(C)** Heatmap of the expression of DE genes identified between 1G12 and EV by all samples are shown. **(D)** Gene Ontology (GO) pathway analysis showcasing the top 5 featured pathways among genes significantly downregulated in EV+IL-2 Treg cells when compared with EV Tregs. **(E)** Gene Ontology (GO) pathway comparison analysis showcasing the top five featured pathways among genes significantly downregulated in 3A10t samples when compared with EV samples. In D and E, the plots depict the gene ratio of DE genes in each pathway, with the circle size indicating the gene count of DE genes in each pathway and the color representing the adjusted P values.

Pair-wised comparison with EV no IL-2 revealed hundreds of differentially expressed genes (DEGs) in EV+IL-2 and 3A10t Tregs, but 1G12t had very few DEGs (Fig. 5, B and C; and Fig. S4, A–C). Two of the upregulated genes in 1G12t Tregs were *IL2Rβ* and *IL2* (Fig. 5 C and Data S1), demonstrating successful transduction of these samples. Notably, both EV+IL-2 and 3A10t, but not 1G12t, had increased expression of *Socs2* and *Cish*, known negative feedback regulators of IL-2 signaling. These data did not reveal strong negative feedback control of IL-2 signaling in 1G12t Tregs. The similar transcriptomic profile between 1G12t Tregs and Tregs deprived of IL-2 suggested a block of 1G12t signaling upstream of gene expression. pSTAT5 analyses in Fig. 4 used Tregs on day 3 after transduction and the RNA-seq experiment used Tregs on day 7 after transduction. It was possible that 1G12t Tregs activated strongly early after transduction but then adapted through epigenetic changes during the 7-day period. We thus analyzed pSTAT5 as soon as the transgene expression was detectable between 15 and 18 h after transduction. At all time points, pSTAT5 signal was significantly lower in 1G12t Tregs than in 3A10t Tregs, indistinguishable from that in EV transduced cells (Fig. S3 E). Taken together, the low IL-2 signaling in 1G12t Tregs was not likely due to cellular adaptation to high-affinity interactions between 1G12 and orthoIL-2R. It is possible that 1G12 engagement with its ortho receptor shortly after synthesis led to ligand-induced receptor endocytosis and degradation of 1G12t.

The transcriptomic data of EV+IL-2 and 3A10t groups presented an opportunity to explore differential transcriptomic impact of soluble versus tethered IL-2 signaling in Tregs. Both groups showed increased IL-2 signaling indicated by upregulation of *Socs2*, *Cish*, *Bcl2*, *Lta*, and *Tnfrsf9* expression (Fig. 5 D). Expression of some Treg suppression-associated molecules (*IL10*, *Ebi3*, *IL12a*, *GzmB*, and *Prf1*) increased, whereas others were unchanged (*Ctla4*, *Tgfb1*, and *Flg2*) or decreased (*Nt5e* and *Entpd1*) when compared with EV without IL-2 (Fig. 5 D). Pathway analysis of the upregulated DEGs in the two groups revealed shared cellular programs in cytoplasmic translation (*Rpl10*, *Rpl15*, and *Rps6*). 3A10t showed stronger enrichment for metabolic genes (*Cox5a*, *Ndufa10*, *Chchd2*, and *Uqcrh*), whereas EV+IL-2 had stronger activation of cell cycle (*Birc5*, *Ccnd2*, *Ccnd3*, *Cdk1*, and *Psrc1*) and type II IFN response genes (*Ifitm1*, *Ifitm3*, and *Gbp4*) (Fig. 5, D and E; and Fig. S4 D). Selectively downregulated genes in 3A10t Tregs were enriched in apoptosis- and cell cycle checkpoint-pathways (*Bcl2l11*, *Tnfrsf1b*, *Stk24*, *Wac*, and *Ppp2r5c*) (Fig. 5 E and Fig. S4 E). Together, these transcriptomic analyses suggested that 3A10t promoted mitochondrial metabolism and suppressed apoptosis in Tregs, which might explain its efficacy in sustaining Tregs in the absence of IL-2.

### Tethered orthoIL-2 3A10 endowed Tregs autonomy from exogenous IL-2 and superior *in vivo* suppressive activity

**Figure 6.**
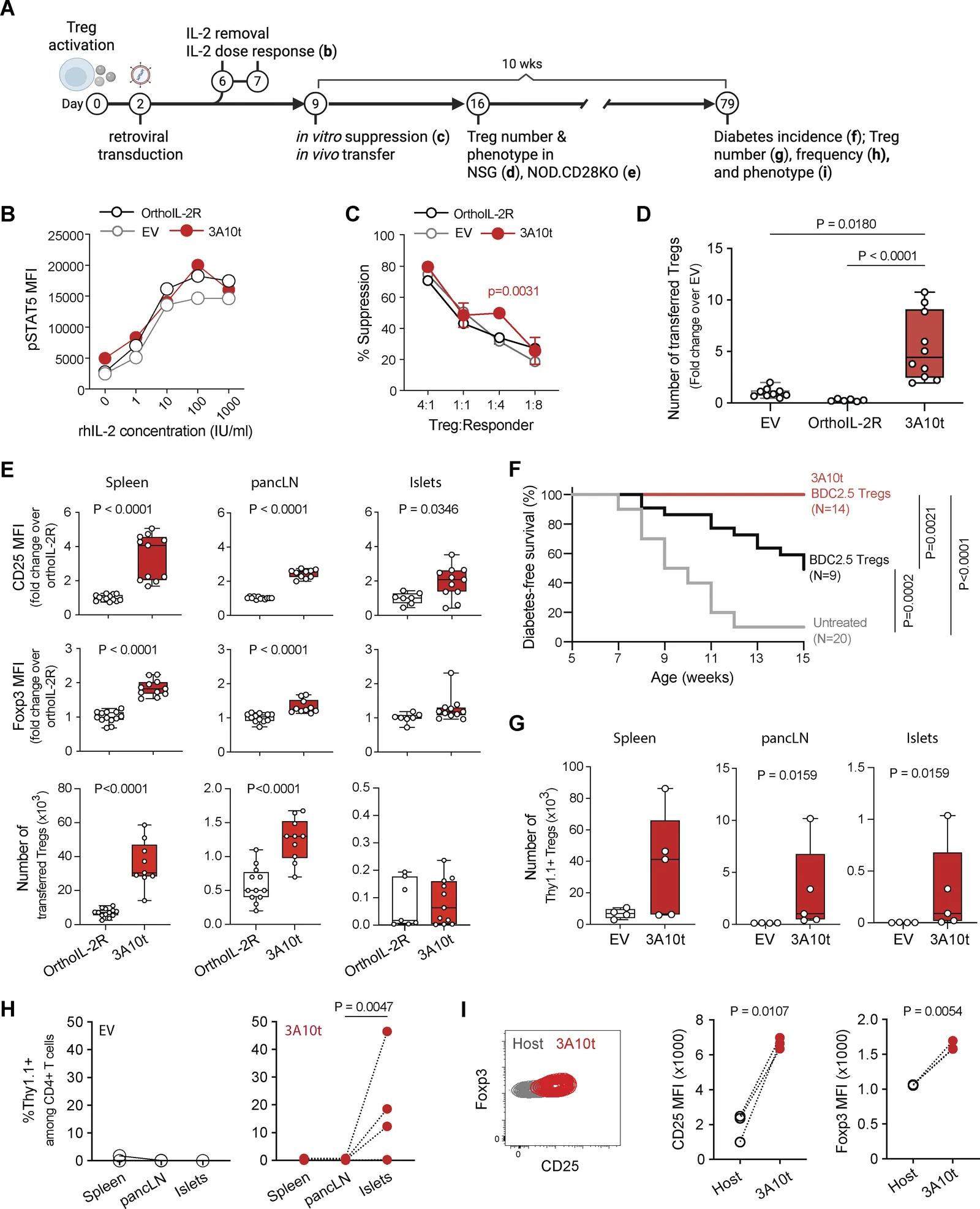
**Function of Tregs expressing tethered 3A10-orthoIL-2R. (A)** Experimental workflow for assessing function of 3A10 expressing Treg *in vitro* and *in vivo*. Created in BioRender. Tang (2026) https://BioRender.com/hloc6vm. **(B)** pSTAT5 in response to titrated concentrations of exogenous IL-2 in Tregs transduced with EV, orthoIL-2R, or 3A10t. Results shown are representative of two independent experiments. **(C)***In vitro* suppression. Ordinary two-way ANOVA followed by Tukey’s multiple comparison after test was used to determine the statistical significance of the difference observed. P values on the graph are for comparisons between 3A10t^+^ and orthoIL-2R^+^ Tregs at each Treg:responder ratio. **(D)** NSG mice were injected with NOD Tregs transduced with EV, orthoIL-2R, or 3A10t. The numbers of transferred Tregs in the spleens of the recipient mice were analyzed 1 wk after cell injection. Numbers shown were normalized to EV control. Data shown were a summary of three independent experiments (means ± SD, *n* = 8–10 mice per group). Kruskal–Wallis test was used to determine the statistical significance among the groups. **(E)** NOD.CD28KO mice were injected with 20,000 FACS-purified Thy1.1^+^ orthoIL-2R or 3A10t-transduced BDC2.5 Tregs. CD25 and Foxp3 expression (normalized to orthoIL-2R control) in the transferred Tregs and the numbers of transferred Thy1.1^+^ Tregs in the spleens, pancLN, and islets were analyzed 1 wk later. Data shown are a summary of three independent experiments (*n* = 7–13 mice per group). Mann–Whitney test was used to determine the statistical significance. **(F)** NOD.CD28KO mice were treated with either 2,000 EV- or 3A10t-transduced BDC2.5 Tregs. Mice with two readings of blood glucose >250 mg/dl were considered diabetic (*n* = 14–28 mice per group in two experimental replicates). Statistical significance was determined using Kaplan–Meier survival analysis. P values were calculated using Mantel–Cox test comparing all experimental groups. **(G–I)** NOD.CD28KO mice were injected with 2,000 FACS-purified Thy1.1^+^ EV- or 3A10t-transduced BDC2.5 Tregs and analyzed 10 wk later. **(G)** Numbers of transferred Thy1.1^+^ Tregs recovered in the spleens, pancLN, and islets. Mann–Whitney test was used to determine the statistical significance. **(H)** Percentages of Thy1.1^+^ Tregs among CD4^+^ T cells. Friedman test with Dunn’s multiple comparison test were used to determine statistical significance. **(I)** Expression of Foxp3 and CD25 in endogenous Tregs and transferred Thy1.1^+^ Tregs in the islets. Paired *t* test was used to determine the significance of the differences. For all panels, only statistically significant P values are listed.

**Figure S5.**
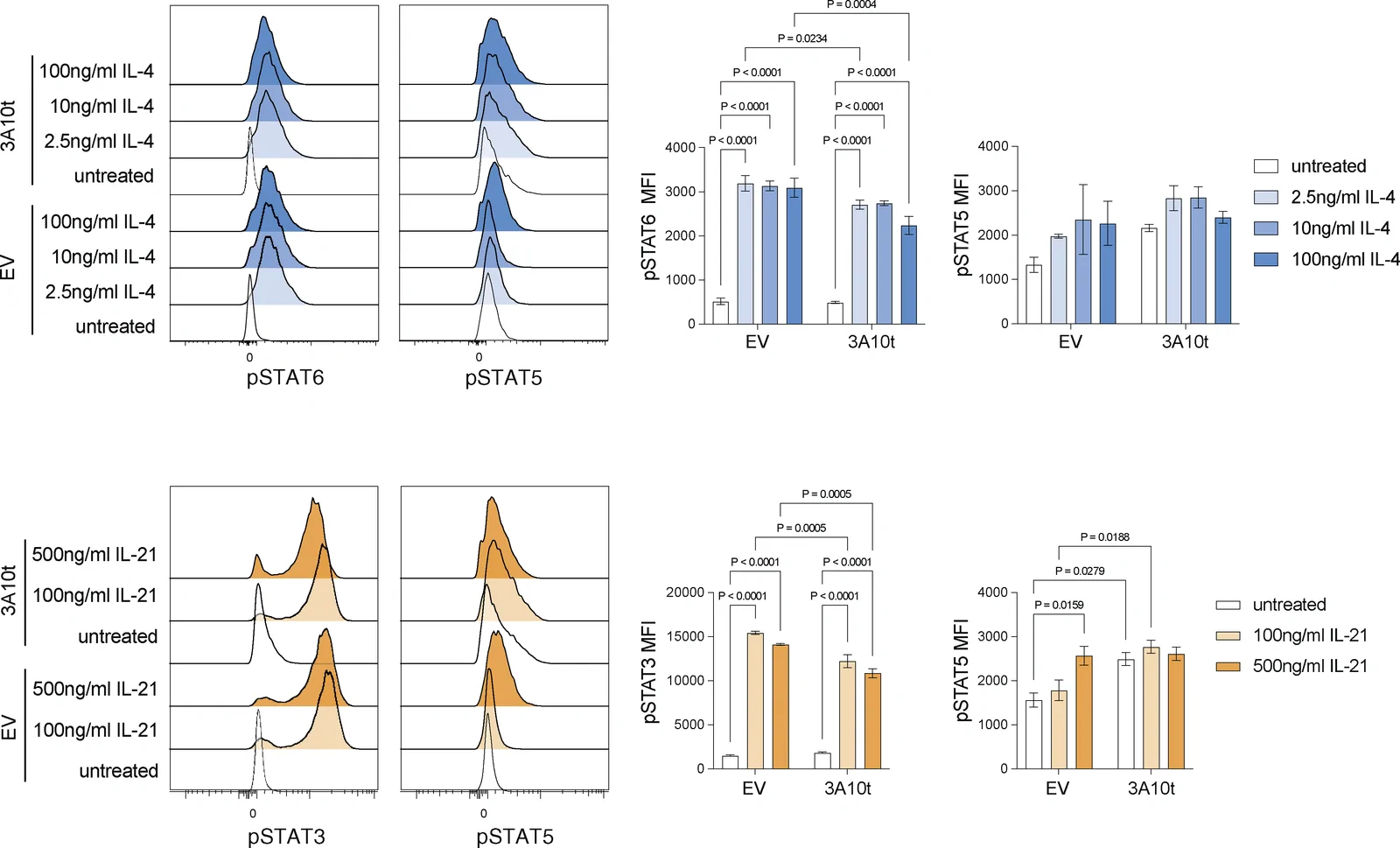
**Response of 3A10t Tregs to IL-4 and IL-21 (related to Fig. 6).** 3A10t- and EV-transduced Tregs were washed and cultured in IL-2–free media overnight. The cells were then stimulated with the indicated concentrations of IL-4 (upper panels) or IL-21 (lower panels) for 30 min, followed by flow cytometric analysis for pSTAT3, pSTAT6, and pSTAT5. Representative flow cytometry plots are shown on the left and data summary are shown on the right. Statistical significance of the difference was determined using two-way ANOVA followed by Tukey’s multiple comparison test.

To determine if autocrine signaling through 3A10t would alter the ability of 3A10t-expressing Tregs to sense and consume exogenous IL-2, we measured pSTAT5 signaling in response to titrated concentrations of IL-2 (Fig. 6 A). Dose response of 3A10t-engineered Tregs was similar to that of EV-transduced and orthoIL-2R–transduced Tregs (Fig. 6 B). To determine if 3A10t-engineered Tregs could respond to other common γ chain-dependent cytokines, we compared the responses of 3A10t- and EV-transduced Tregs with IL-4 and IL-21. The results show 3A10t Tregs retained their ability to respond to these cytokines by increasing STAT6 and STAT3 phosphorylation, respectively (Fig. S5). Moreover, we observed similar *in vitro* suppression when compared with Tregs expressing the orthoIL-2R alone (Fig. 6 C). These results suggest 3A10t-engineered Tregs retained their ability to sense and consume wtIL-2, which is important for their suppressive function.

A significantly higher number of 3A10t-transduced Tregs were recovered 1 wk after transferring to NSG mice when compared with EV- or orthoIL-2R–transduced Tregs (Fig. 6 D). In NOD.CD28KO mice, we observed increased CD25 and Foxp3 expression on 3A10t-engineered BDC2.5 Tregs and increased cell number in the spleens and pancLN (Fig. 6 E). BDC2.5 Tregs transduced with 3A10t had significantly higher CD25 expression than orthoIL-2R–transduced Tregs in the islets (Fig. 6 E). A single infusion of 2,000 3A10t-engineered BDC2.5 Tregs completely prevented diabetes in NOD.CD28KO mice (Fig. 6 F). At 10 wk after transfer, the numbers of 3A10t Tregs recovered from spleen, pancLN, and islets were variable but generally higher when compared with EV Tregs (Fig. 6 G). However, 3A10t Tregs were <1% among the CD4^+^ T cells in the spleens and pancLN, whereas higher percentages were seen in the pancreatic islets (Fig. 6 H). These data suggest that the effect of 3A10t on BDC2.5 Tregs was most pronounced in the antigen-rich environment of inflamed islets. Previously, we have shown that Tregs in the inflamed islets had lower CD25 and Foxp3 expression secondary to a deficiency in IL-2 (Tang et al., 2008). We thus measured the expression of CD25 and Foxp3 on 3A10t Tregs recovered from the islets 10 wk after transfer and observed significantly higher expression of both molecules on 3A10t Tregs when compared with endogenous Tregs (Fig. 6 I).

### Inserting tethered orthoIL-2 into Foxp3 locus safeguards against toxicity of off-Treg expression

**Figure 7.**
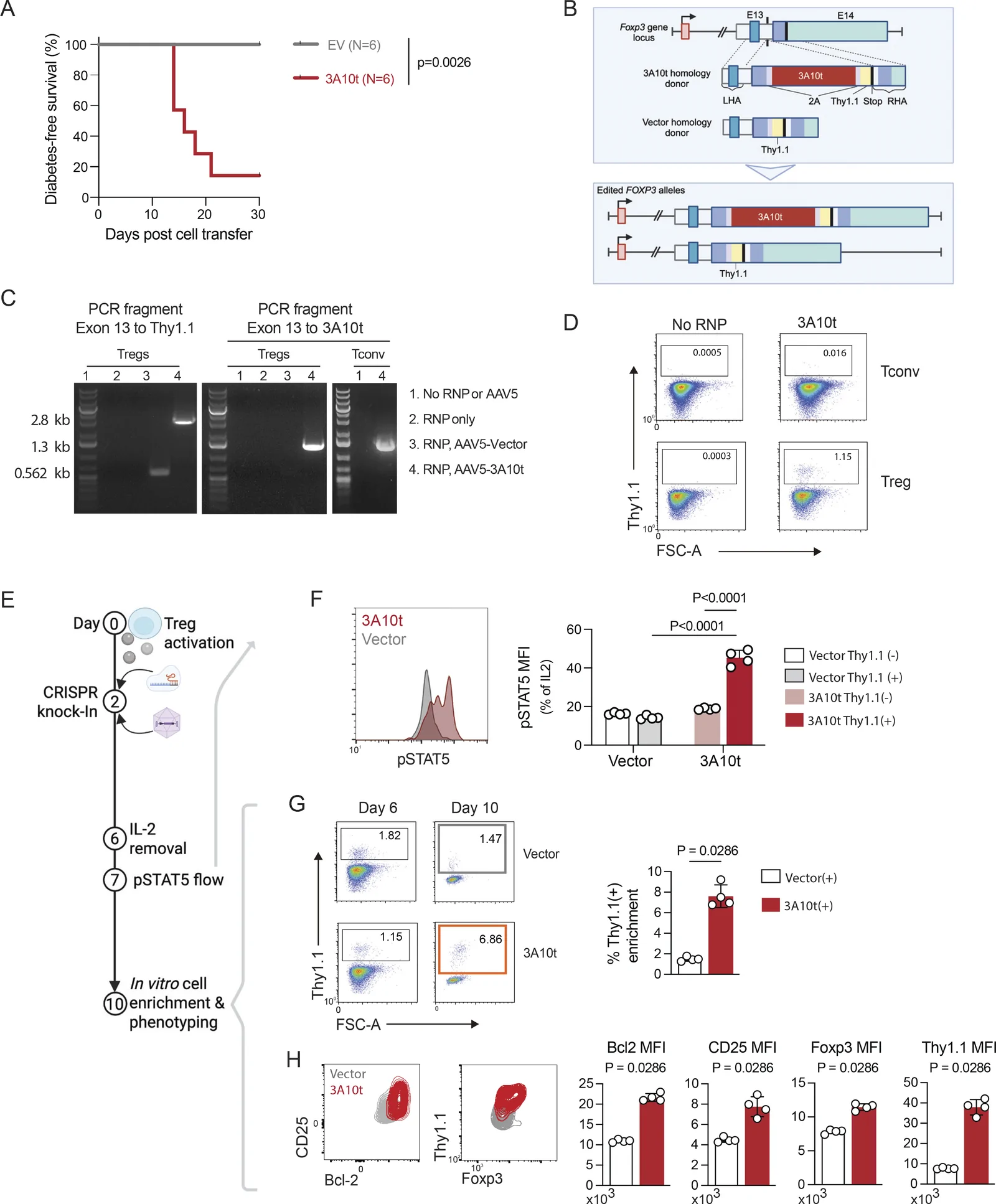
**Safe engineering of the tethered orthoIL-2–IL-2R system. (A)** Diabetes development in NOD.CD28KO mice that received 20,000 CD4^+^ BDC2.5Tg TCR transgenic T cells transduced with EV or 3A10t. Results shown are a summary of two independent experiments. Statistical significance was determined using Kaplan–Meier survival analysis. P values were calculated using Mantel–Cox test. **(B)** Schematic of 3A10t and vector knock-in strategy. Created in BioRender. Tang (2026) https://BioRender.com/nq4hkzh. **(C)** PCR of genomic DNA showing vector and 3A10t insertion into the Foxp3 locus. Uncropped gel images are available online. **(D)** Flow cytometric analysis of Thy1.1 expression in Tregs and Tconvs 4 days after editing, day 6 after activation. **(E)** Experimental workflow for evaluating the function of 3A10t Foxp3 knock-in in NOD Tregs. **(F)** pSTAT5 1 day after IL-2 withdrawal. **(G)** Fold change Thy1.1^+^ Treg percentages cultured 4 days without exogenous IL-2 from day 6 to day 10 after activation. The 3A10t Tregs in this panel are the same as the 3A10t Tregs shown in D. **(H)** Expression of Bcl2, CD25, Foxp3, and Thy1.1 by Thy1.1^+^ cells in vector knock-in and 3A10t knock-in Tregs cultured without IL-2. Statistical significance was determined using Mann–Whitney test. Only statistically significant P values are shown. Source data are available for this figure: SourceData F7.

Our results thus far showed that a tethered orthoIL-2–IL-2R system could sustain Tregs in the absence of exogenous IL-2. To determine if this system could also potentiate proinflammatory Tconvs, we expressed 3A10t in islet-specific CD4^+^ Tconv and assessed their diabetogenic potential in young prediabetic NOD.CD28KO mice. BDC2.5 Tconv cells expressing the 3A10t construct accelerated diabetes development when compared with EV-transduced BDC2.5 Tconvs (Fig. 7 A).

To ensure the selective expression of the orthoIL-2–IL-2R system in Tregs, we targeted the 3A10t construct to the Foxp3 locus by inserting it in the last intron of the *Foxp3* gene (Fig. 7 B). PCR of genomic DNA from edited cells showed successful knock-in of the vector and the 3A10t constructs in both Tregs and Tconvs (Fig. 7 C). Thy1.1 could be detected on the edited Tregs, not on Tconvs, demonstrating Treg-selective expression of the construct (Fig. 7 D).

To determine if the expression of 3A10t driven from the Foxp3 locus could support Tregs in the absence of exogenous IL-2, we removed IL-2 from the Treg cultures and tested the cells using a battery of *in vitro* assays (Fig. 7 E). Tregs with 3A10t knock-in exhibited higher pSTAT5 MFI (Fig. 7 F) and showed selective survival advantage after IL-2 withdrawal (Fig. 7 G). 3A10t knock-in Tregs had higher expression of Bcl2, CD25, and Foxp3 (Fig. 7 H). Intriguingly, we observed significantly higher Thy1.1 expression on 3A10t knock-in Tregs, but not in vector knock-in Tregs, suggesting a positive feedback loop of IL-2 signaling enhancing Foxp3 and 3A10t-Thy1.1 expression (Fig. 7 H). These results show that knocking the 3A10t construct into the Foxp3 locus could achieve Treg-specific expression and functional support of Treg autonomy with the added benefit of positive feedback loop reinforcing autocrine receptor expression and Treg lineage identity.

## Discussion

Although Tregs constitutively express the high-affinity trimeric IL-2R complex, delivering IL-2 selectively to Tregs remains an elusive goal due to the pleotropic effects of IL-2 on other IL-2–responsive cells besides Tregs. The ortho autocrine IL-2–IL-2R system developed in this study provided an effective solution to address this challenge to Treg therapy. This system sustained Treg persistence independent of exogenous IL-2, which can be limiting in diseased settings (Driver et al., 2012; Tang et al., 2008; Yamanouchi et al., 2007; Yang et al., 2022; Yshii et al., 2022). The enhanced proliferation and survival of Tregs expressing the autocrine orthoIL-2–IL-2R allowed the infused Tregs to establish persistent numerical dominance over inflammatory cells without aberrant accumulation or systemic immunosuppression. This approach also led to increased expression of CD25 and Foxp3, molecules important for Treg function and lineage stability.

Two other cell-engineering approaches for enabling IL-2 signaling autonomy in Tregs have been reported. One approach used a chemically inducible signaling complex (CISC), which allows temporally controlled IL-2 signaling selectively in engineered cells using rapamycin (Cook et al., 2023). The other approach engineered Tregs to secrete a partial agonist of human IL-2 (hIL-2pa), enabling highly efficient *in vivo* expansion of the engineered Tregs (Robert et al., 2025). The number of engineered Tregs persistently increased over a 6-mo period after adoptive transfer so that they constituted 15% of the CD4^+^ T cell compartment and over 60% of the Treg compartment systemically. The ortho autocrine IL-2–IL-2R system described in this study similarly improved the efficacy of therapeutic Tregs as described in these previous reports. This system is selective for the engineered Tregs as the CISC system, but different by being cell autonomous and not reliant on the exogenous provision of an inducer. Our study and the hIL-2pa report both show superior efficacy of weak IL-2 agonists when compared with wtIL-2. However, the 3A10t molecule in our study was ortho to the engineered cells with no discernable impact on the phenotype or the numbers of endogenous Tregs. Long-term follow-up showed that this tethered orthoIL-2 system did not lead to systemic dominance of the engineered Tregs, and the most pronounced effect was localized to the pancreatic islets, where the cognate antigen for the BDC2.5 Tregs was most abundant.

It has been previously shown that diabetes protection by adoptively transferred Tregs requires islet antigen-specific Tregs (Tang et al., 2004; Tarbell et al., 2004). BDC2.5 Tregs protected against autoimmune diabetes by inhibiting LN priming of effector T cells (Tang et al., 2006), collapsing CD4^+^ and CD8^+^ effector program in inflamed islets partially through IL-2 deprivation (Mahne et al., 2015), followed by a gradual reduction of number and the inflammatory phenotype of the myeloid cells in the islets due to reduced recruitment and IFNg (Klementowicz et al., 2017). Our RNA-seq analysis showed that the main effect of 3A10t expression was enhanced mitochondrial respiration and decreased apoptosis without marked alteration to Treg suppressive programs. We speculate that mechanisms of action of 3A10t Tregs are likely similar to those reported previously using unmodified BDC2.5 Tregs. The improved efficacy of 3A10t-engineered Tregs is likely twofold—enhanced proliferation and survival providing a numerical advantage to the infused Tregs and higher expression of CD25 enabling more efficient competition for IL-2 to subvert effector T cell function. It remains to be determined how long BDC2.5 Tregs need to persist to maintain their therapeutic effect. It has been previously reported that long-term diabetes protection by BDC2.5 Tregs was due to infectious tolerance (Tarbell et al., 2007).

It is important to note that 3A10t-engineered BDC2.5 Tregs constituted only a very small percentage of the T cell compartment in the spleens and pancLNs. This small increase of Tregs is unlikely to cause systemic immunosuppression. The strong efficacy in diabetes prevention by 3A10t engineered BDC2.5 Tregs is likely due to their improved fitness in the inflamed islet tissue. Polyclonally expanded Tregs have been shown to be safe in type 1 diabetes, but efficacy has not been clearly demonstrated (Bender et al., 2024; Bluestone et al., 2015; Marek-Trzonkowska et al., 2016), likely due to insufficient islet specificity. Tregs can be engineered to express islet antigen-specific TCRs (Porret et al., 2026; Yang et al., 2022) or CARs (Obarorakpor et al., 2023; Pieper et al., 2024, *Preprint*; Spanier et al., 2023) to direct their function to pancreatic islets. The IL-2 and islet antigen-specificity engineering modules will likely be synergistic to achieve durable efficacy in restoring tolerance to pancreatic islets. In this regard, it has been shown that combining CISC-induced IL-2 signaling with islet antigen-specific TCR promoted proliferation of engineered Tregs in preclinical models (Uenishi et al., 2024). Encouragingly, a clinical trial of CISC and TCR dually engineered Tregs is open and currently recircuiting patients (NCT06919354).

The cellular and molecular impact of autocrine IL-2 signaling in engineered Tregs requires more in-depth investigations. Although it is generally accepted that Tregs do not produce IL-2, thus only rely on paracrine IL-2 signaling, this dogma has been challenged in a recent report suggesting a role of Treg-intrinsic IL-2 in Treg generation and maintenance (Chawla et al., 2020). Previously, autocrine IL-2 was shown to induce weaker STAT5 signaling that limited effector differentiation in favor of a memory cell fate in CD8^+^ T cells (Kahan et al., 2022). Pre-assembly of the IL-2R complex can occur in the ER and Golgi in autocrine cells (Volko et al., 2019), thus autocrine IL-2-IL-2R signaling could be initiated intracellularly, whereas paracrine signaling could only be initiated at the cell surface. This distinction may lead to differential engagement of signaling molecules that are responsible for distinct cellular behaviors.

Taken together, this study presents a cell-engineering solution to promote Treg fitness and persistence in a cell autonomous fashion. The constructs developed in this study are based on mouse protein sequences; thus, they do not work in human Tregs. We are currently developing an ortho autocrine IL-2 system for human Tregs. Moreover, future studies are needed to fine-tune cell engineering approaches optimize efficacy and safety of this system. For example, we have shown that the autocrine IL-2 can exacerbate autoimmune pathology if the expression is not limited to Tregs. Inserting the construct into the endogenous Foxp3 locus was effective in managing this risk for mouse cells. However, this approach may not work as well in human Tregs since FOXP3 expression is not limited to Tregs in humans. Driving the expression off a synthetic Treg-specific promoter may be a safer approach. Additionally, persistent IL-2 signaling could drive excessive Treg proliferation, raising concern of oncogenesis or systemic immunosuppression. Although this was not seen with 3A10t-engineered Tregs, this risk should be thoroughly investigated in human Tregs with autocrine IL-2. This may be managed by using an inducible or oscillating expression system for periodic IL-2 stimulation or by engineering a kill switch to delete the cells if warranted. The design and testing principles from this study can guide the development of a safe and effective autocrine IL-2 system for human Tregs for clinical translation.

## Materials and methods

### Animals

NOD/ShiLtJ (Jackson Laboratories), NOD.Thy1.1, NOD.CD28KO (Salomon et al., 2000), NOD.BDC2.5 TCR transgenic, and NOD-scid IL-2Rgamma^null^ (NSG, Jackson Laboratories) mice were housed and bred in accordance with the University of California, San Francisco (UCSF) Institutional Animal Care and Use Committee guidelines. Littermates were used as controls when possible, and all animals were age-matched in diabetes studies. NOD.Thy1.2 and NOD.Thy1.1 mice, 7–14 wk, were used as donors of Treg and naive CD4^+^ T cells. NSG mice were used as recipients at ages 7–14 wk for experiments. NOD.CD28KO mice were used as recipients in experiments starting at 5–6 wk of age. Mice used in experiments were randomized based on age and sex so that these variables are equally distributed among experimental groups. All mouse experiments were performed according to a UCSF Institutional Animal Care and Use Program–approved protocol (IACUC protocol no. AN200668).

### Cell culture media

Cells were cultured in DMEM containing 4.5g/l glucose, supplemented with 10% heat-inactivated fetal calf serum, 100 IU/ml penicillin and streptomycin, 10mM of HEPES, GlutaMax, sodium pyruvate, nonessential amino acids, and 50 μM β-mercaptoethanol. Cells were resuspended in PBS supplemented with 2% heat-inactivated fetal calf serum for isolation, enrichment, and sorting. Vendor and catalog information for key cell culture reagents can be found in Table S1.

### Mouse Treg isolation and expansion

Single-cell suspensions were prepared from lymph nodes and spleens from NOD or NOD.BDC2.5 TCR transgenic mice. CD4^+^ T cells were enriched using a magnetic negative selection kit followed by purification of CD4^+^CD8^−^CD62L^+^CD25^+^ Tregs and CD4^+^CD62L^+^CD25^−^ naïve Tconvs using fluorescence activated cell sorting (FACS) on a BD Aria2. Flow cytometric antibodies used are summarized in Table S2. FACS-purified Tregs were stimulated with CD3/CD28 dynabeads at the ratio of 3:1 (beads:Treg) and cultured in cell culture media supplemented with 2,000 IU/ml rhIL-2. FACS-purified Tconvs were stimulated at 1:1 bead to cell ratio and cultured in cell culture media supplemented with 200 IU/ml rhIL-2. The cells were counted and split every 2–3 days until used in experiments.

### Retroviral constructs and T cell transduction

Retroviral constructs were cloned into an MSCV vector containing a mouse Thy1.1 reporter behind an internal ribosomal entry site (IRES). The orthoIL-2R and various tethered IL-2–IL-2R constructs were cloned upstream of the IRES. Retroviral Plat-E packaging cells (Table S1) were transfected using Lipofectamine 2000 with the MSCV plasmids, and virus was collected 48 h later and used with or without Retro-X concentrator. On day 2 after activation, T cells were spinfected for 90 min at 25°C 600 *g* in the presence of 20 μg/ml of polybrene. Following spinfection, virus was removed, and cells were resuspended in culture media with or without 2,000 IU/ml rhIL-2, as indicated in the result section and figure legends. Key molecular biology reagents used are summarized in Table S3. Amino acid sequences of all constructs are listed in Table S4.

### Analytical flow cytometry

Transduced Treg cultures were washed to remove rhIL-2 immediately after transduction or on day 2 after transduction, as indicated in the result section and figure legends. The cells were rested for 15–18 h in culture medium without exogenous IL-2. In experiments testing soluble IL-2, the rested cells were stimulated 30 min with titrated concentrations various mouse IL-2 for pSTAT5 analyses. For experiments testing Tregs with receptor tethered IL-2, overnight rested Tregs were analyzed for pSTAT5, pSTAT3, and pSTAT6 without stimulation or after 30-min stimulation with titrated concentrations of rhIL-2, mouse IL-4, or mouse IL-21 as indicated. For Akt phosphorylation, engineered Tregs were rested without exogenous IL-2 from day 2–4 after transduction. As a positive control, 2,000 IU/ml of rhIL-2 was added to the Tregs engineered with orthoIL-2R without a tethered ligand for varying durations as indicated. For pSTAT5, pSTAT3, pSTAT6, and pAKT analyses, cells were fixed in 4% paraformaldehyde for 10 min and permeabilized with 100% ice-cold methanol for 30 min. Cells were stained with antibodies to Thy1.1 and various phosphoproteins. For cell surface and Foxp3 staining, cells were stained for extracellular surface markers for 30 min on ice, fixed with a Foxp3 transcription factor fixative for 30 min on ice, permeabilized, and stained for intracellular markers for 30 min to 1 h on ice. Information for all antibodies and reagents used are summarized in Table S2. All samples were run on a BD LSRII flow cytometer.

### *In vitro* T cell proliferation assay

NOD Tregs were transduced and cultured for 10 days, washed to remove exogenous IL-2, and rested for 18 h. Cells were stained with Cell Tracker Violet (CTV, Table S2) and restimulated with CD3/CD28 dynabeads (1:1). Cells were assessed 72–96 h later for CTV dilution.

### *In vitro* suppression assay

Transduced Tregs from NOD.Thy1.2 mice were FACS purified based on Thy1.1 expression on day 10 after activation and rested for 6 h. Naive CD4^+^CD62L^+^CD25^−^ cells from NOD.Thy1.1^+^ T cells were FACS purified and stained for CTV. Thy1.2^+^Thy1.1^+^-transduced Tregs and naive Thy1.2^−^Thy1.1^+^ responder CD4^+^ T cells were co-cultured at various ratios and stimulated with plate-bound 0.5 μg/ml anti-CD3 and 1 μg/ml anti-CD28. Proliferation of the responder T cells was measured on day 3 after stimulation using CTV dilution. Percent suppression was calculated using the following formula: (% CTV^lo^ without Tregs − %CTV^lo^ with Tregs)/%CTV^lo^ without Tregs.

### *In vivo* experiments

For experiments in NSG mice, FACS-purified Thy1.2^+^ NOD Tregs were activated, transduced, and a mixture of transduced Thy1.1^+^, and non-transduced Thy1.1^−^ cells were infused via retro-orbital injection. For experiments in NOD.CD28KO mice, Thy1.2^+^ BDC2.5 Tregs were activated and transduced, and Thy1.1^+^ transduced Tregs were FACS purified before *in vivo* transfer via retro-orbital injection. MSA-fused orthoIL-2 and MSA-fused mouse wtIL-2 were injected i.p. in 100 μl volume. Diabetes development in NOD.CD28KO mice was assessed by measuring blood glucose. Mice with two readings of blood glucose higher than 250 mg/dl were considered diabetic.

### Tethered IL-2–IL-2R structure modeling

The predicted structure of tethered orthoIL-2–IL-2R was generated with AlphaFold protein structure databases. The predicted structure was then modeled with PyMOL, a molecular visualization software (https://www.pymol.org/). Each protein is colored differently for recognition.

### RNA-seq

Purified mouse Tregs were transduced with either 1G12t, 3A10t, or EV as a control on day 2 after activation. The cells were washed to remove exogenous IL-2 on day 4 (2 days after transduction) and cultured for 5 additional days. Half of the EV-transduced culture was left in 100 IU/ml recombinant mouse IL-2–containing medium as a positive control. The transduced cells were FACS purified based on the expression of the Thy1.1 on day 9. Cells were lysed using TCL buffer (Qiagen), and RNA was extracted using VAHTS RNA Clean Beads (Vazyme), then eluted in 10 μl of nuclease-free water. cDNA synthesis and amplification were performed using the Discover-sc WTA kit version 2 (Vazyme) according to the instruction provided by the manufacture. The amplified cDNA products were purified using VAHTS DNA Clean Beads (Vazyme) and quantified using Qubit. Illumina sequencing libraries were made using 50 ng of the amplified cDNA using the TruePrep DNA Library Prep Kit version 2 for Illumina (Vazyme) and the TruePrep Index Kit version 2 for Illumina (Vazyme). Size selection of the PCR product was performed using 0.55× VAHTS DNA Clean Beads. Finally, 20 ng of each sample were pooled for subsequent second-generation sequencing on the Illumina NovaSeq X, with a read length of 50 bp for paired end. Key molecular biology reagents used are summarized in Table S3. RNA-seq data are openly available at GEO (GSE285527).

Raw fastq reads were trimmed by Cutadapt (version 1.18) to trim adapter and low-quality sequence. Reads were aligned to the mouse genome (mm10) using STAR (version 2.5.3). The number of reads within each gene in each sample were counted using RSEM (version 1.3.0) with gene annotation file from GENCODE (GRCm38.m23). Differential expression was estimated by using DESeq2 package (version 1.26). DEGs in EV+IL-2, 3A10t, and 1G12t Treg in comparison with EV without IL-2 Tregs were defined as log2 fold change >0.25 and P value <0.01. Pathway analysis was performed by using the cluster Profiler R package (version 3.14.3) with default parameters (P value <0.05) and the functional annotations terms in Gene Ontology (Yu et al., 2012).

### Adeno-associated virus (AAV) production

The fragments LHA-oExon14-P2A-Thy1.1-Stop-RHA and LHA-oExon14-T2A-3A10t-P2A-Thy1.1-Stop-RHA were cloned into AAV2-ITR transfer plasmids. These transfer plasmids, along with pAAV2/5 Rep-Cap plasmids and adenovirus helper plasmids, were transfected into HEK293T cells using polyethylenimine to package the donor template into AAV5 capsids. On day 3 after transfection, cells were scraped and collected in AAV lysis buffer (50 mM Tris and 150 mM NaCl). The cells were lysed using three rounds of freeze and thawing, followed by a 1-h incubation at 37°C with 25 units/ml Benzonase (#70-664-3; Millipore Sigma). The AAV particles were purified using iodixanol gradient ultracentrifugation (OptiPrep, STEMCELL Technologies). The iodixanol layer between 40 and 54% was extracted using an 18-gauge needle. The AAV was washed and concentrated using storage buffer (1X PBS with 0.001% Tween-20).

### CRISPR knock in

Lyophilized gRNA (5′-AGC​CUG​GGG​CUA​GAC​AUG​UG-3′, IDT) was resuspended in nuclease-free TE buffer at a concentration of 100 μM, aliquoted, and stored at −80°C. Recombinant Cas9-NLS (40 μM) was purchased (QB3 MacroLab). For each reaction, an equal molar mixture of gRNA and Cas9 (60 pmol each) was incubated at room temperature for 15 min to form ribonucleotide-protein complexes (RNP). Dynabeads were removed from activated Treg and Tconv cells on day 2 after activation using magnetic separation. For each electroporation reaction, 200,000 cells were resuspended in 20 μl Lonza electroporation buffer P3 and mixed with 3 μl of RNP and electroporated in a Lonza 4D 96-well electroporation system using pulse code DN100 (Treg) or EH115 (Tconv). After electroporation, 80 μl of pre-warmed culture medium was immediately added to the electroporated cells, and the cells were rested for 10 min at 37°C. The rested cells were seeded in wells of 96-well round-bottom plates in 200 μl culture medium and 20 μl AAV virus. Cells were subsequently cultured and maintained at a density of 1 million cells/ml.

### Statistics

Statistical analyses were performed with the GraphPad Prism 9 software. In diabetes protection experiments with prior knowledge of the incidence of untreated mice at 80–90%, power calculations were performed. In general, minimally eight mice per experimental group are needed to detect a significant reduction of diabetes incidence by 50%. When no power calculation was possible due to a lack of prior data, three to five biological replicates were included per experimental condition, and all results shown were repeated at least once in an independent experiment with similar results. Mann–Whitney test was used to determine the statistical significance between the two experimental groups. Ordinary one-way ANOVA Kruskal–Wallis test followed by Dunn’s multiple comparison test was used to determine statistical significance among three or more experimental groups with one independent variable. Ordinary two-way ANOVA followed by Tukey multiple comparison test was used to determine statistical significance among three or more experimental groups with two independent variables. Statistical significance of diabetes-free survival was determined using Kaplan–Meier survival analysis and using Mantel–Cox test to calculate P values comparing each condition to BDC2.5 Treg-treated group.

### Online supplemental material

Fig. S1 contains additional original data in support of Fig. 2. Fig. S2 contains additional original data in support of Fig. 3. Fig. S3 contains modeling and additional original data in support of Figs. 4 and 5. Fig. S4 contains additional original data in support of Fig. 5. Fig. S5 contains additional original data in support of Fig. 6. Tables S1, S2, and S3 contain information of key reagents used in the study. Table S4 contains amino acid sequences of constructs developed in this study. Data S1 contains source data for Fig. 5 C.

## Supplementary Material

Table S1 shows key cell culture reagents.

Table S2 shows key molecular biology reagents.

Table S3 shows amino acid sequences of various IL-2 and IL-2R constructs.

Table S4 shows flow cytometry reagents.

Data S1 shows contains source data for Fig. 5 C.

SourceData F5 is the source file for Fig. 5.

SourceData F7 is the source file for Fig. 7.

## Data Availability

The data underlying all figures are available in the published article and its online supplemental material. RNA-seq data are openly available at GEO (GSE285527).
