# Supplementary material for: Receptor-tethered orthogonal IL-2 enhances regulatory T cell therapy
Source: J Exp Med. 2026 Aug 12;223(9):e20251480. doi: 10.1084/jem.20251480 (PMC13521516; doi:10.1084/jem.20251480)
Supplement: Supplementary file 1 [file jem_20251480_sourcedataf5.pdf]

Source data for Fig 5C

EV+IL-2 DE up genes

Gene	Log2FC	PValue
Lta	1.85	7.69E-44
Ifitm3	3.56	2.20E-42
Qw2b n≥÷S100a6	1.55	1.32E-34
Stmn1	2.95	9.25E-25
Tmco3	1.96	4.01E-24
Hist1h2ap	2.01	4.88E-24
Socs1	1.26	5.39E-22
Lgals1	1.12	2.96E-20
Osm	1.64	1.67E-19
Drc1	1.70	6.42E-19
Socs2	0.86	3.07E-18
Tuba1b	1.19	1.15E-17
Cndp2	1.18	1.56E-17
Rrm2	2.87	1.98E-17
Ifitm2	3.28	2.53E-17
Tap2	1.02	5.91E-17
Dusp6	2.24	8.73E-17
Ly6e	1.12	1.17E-16
Sema4a	1.91	1.64E-16
Prnp	1.10	8.21E-16
Birc5	3.96	1.54E-15
Asf1b	2.34	7.10E-15
Hmgn2	1.37	1.48E-14
Ccnd3	0.99	2.31E-14
Crem	0.99	4.57E-14
Gadd45g	2.10	4.97E-14
Gbp4	1.56	4.88E-14
Cish	0.88	5.19E-14
Serpina3g	2.01	5.92E-14
Cdkn1a	2.00	9.12E-14
Flt3l	1.12	7.82E-13
Xbp1	0.87	1.47E-12
Tnf	1.76	1.54E-12
Cks1b	2.25	1.94E-12
Metrnl	1.83	2.26E-12
Pcx	2.63	2.54E-12
Gata1	1.31	5.30E-12
Rexo2	0.86	5.22E-12
Anxa2	0.89	1.14E-11
Pdlim4	3.25	1.63E-11
Rps27l	0.82	2.58E-11
Elovl6	1.99	3.05E-11
Top2a	3.03	4.88E-11
Stc2	2.17	6.37E-11
Il2rb	0.76	6.80E-11
Gzmb	3.66	7.97E-11
Ifitm1	5.62	1.03E-10
Tasp1	1.33	1.05E-10
Lilrb4a	2.52	1.27E-10
Il2ra	0.75	1.64E-10
Psmb8	0.92	1.72E-10
Vim	0.66	1.74E-10
Trat1	0.73	1.88E-10
Map3k8	1.34	2.00E-10
Tyms	1.72	1.98E-10
Mov10	1.31	3.45E-10
Tmem176b	2.51	4.99E-10
Klhdc2	0.93	6.10E-10
Cdk1	3.35	6.72E-10
Igfbp4	2.22	8.02E-10
Ube2c	3.60	8.32E-10
Aco2	0.84	1.18E-09
Ergic1	1.10	3.39E-09
Actr3b	1.18	5.68E-09
Myc	0.78	5.60E-09
Pigz	2.18	5.70E-09
S100a1	2.11	6.10E-09
Rcn1	1.10	8.07E-09
Hemk1	1.19	1.18E-08
Cdk6	1.40	1.28E-08
H2-Q7	0.86	1.41E-08
H2afy	0.58	2.06E-08
Jaml	1.61	2.13E-08
Ccnb2	3.60	2.51E-08
Cyb5a	0.72	3.47E-08
Actg1	0.57	3.92E-08
Rbbp7	0.72	5.68E-08
Crip1	0.68	7.21E-08
Tnfrsf9	0.93	8.52E-08
Isg20	1.54	8.68E-08
Lilr4b	2.21	1.04E-07
Bax	0.73	1.08E-07
Lgals3bp	1.17	1.35E-07
Hsd11b1	0.81	1.82E-07
Tpm4	0.73	1.89E-07
Phlda3	1.35	2.20E-07
Spc24	3.68	2.88E-07
Ptpn9	1.36	3.03E-07
Tnfaip8	0.87	3.38E-07
Lrrc32	0.86	3.77E-07
Mcm5	1.80	3.79E-07
Hint1	0.69	4.05E-07
H2afz	0.59	4.39E-07
Nkg7	1.78	5.92E-07
Tnfrsf18	0.65	7.23E-07
Tk1	2.49	7.81E-07
Tbca	0.62	7.95E-07
Tspan13	0.71	8.26E-07
Ppm1j	0.91	8.36E-07
Gm10282	1.59	1.02E-06
Tfdp1	1.11	1.12E-06
Bcl2	0.77	1.41E-06
Prelid2	1.22	1.65E-06
Tagln2	0.59	1.89E-06
Slc17a9	0.60	2.01E-06
Trp53i11	0.72	2.18E-06
Glipr2	0.55	2.36E-06
Tcrg-C2	3.98	2.40E-06
Psrc1	2.11	2.59E-06
Rps12	0.67	2.64E-06
Cd200r1	1.08	2.73E-06
Ctsc	0.70	3.47E-06
H2-Q6	0.71	3.50E-06
Ptma	0.48	3.48E-06
Psmb9	0.71	3.56E-06
Rpl15	0.53	4.15E-06
Tcf19	1.52	4.34E-06
Gzmc	2.65	4.60E-06
Ccnd2	0.88	4.76E-06
Arl4c	0.89	5.36E-06
Fsd1l	1.42	5.60E-06
Gmnn	2.11	5.62E-06
Myl10	3.18	5.77E-06
Igtp	0.58	5.99E-06
Junb	0.61	6.22E-06
Tmem176a	2.50	6.20E-06
H2-K1	0.56	8.13E-06
Ndrg1	0.67	8.46E-06
Ccna2	3.70	8.61E-06
Batf	0.69	8.90E-06
Aurkb	2.98	9.24E-06
Cd69	1.28	1.12E-05
Ptpn1	0.64	1.12E-05
Pclaf	3.97	1.15E-05
Dusp1	1.54	1.18E-05
Ptp4a3	0.56	1.40E-05
Tex2	1.53	1.39E-05
Uba52	0.48	1.40E-05
Fam162a	0.59	1.57E-05
Oat	0.65	1.60E-05
D16Ertd472e	0.61	1.62E-05
Sae1	0.56	1.63E-05
Lgals3	0.83	1.69E-05
Calr	0.48	1.94E-05
Rcsd1	0.55	2.03E-05
Arid5a	0.59	2.08E-05
Rpl10	0.47	2.22E-05
Rps5	0.47	2.21E-05
Mir6974	1.69	2.34E-05
Tmem256	0.73	2.35E-05
Kpna2	0.83	2.36E-05
Cdc20	1.56	2.39E-05
Nucb1	0.51	2.49E-05
Ccnb1	2.76	2.52E-05
S100a13	0.62	2.63E-05
Tap1	0.75	3.06E-05
Ran	0.52	3.17E-05
Hmgb2	0.59	3.27E-05
Rps9	0.45	3.35E-05
Pcna	0.62	3.45E-05
Rpl3	0.49	3.60E-05
Fdps	0.85	3.72E-05
Pmf1	1.27	3.75E-05
Rpl4	0.44	3.84E-05
AA467197	2.43	3.89E-05
Ube2s	0.72	4.02E-05
Cd86	1.17	4.57E-05
Slc25a4	0.66	4.57E-05
Ahcyl2	0.65	4.85E-05
Gbp6	1.36	4.82E-05
Trgv2	4.13	4.85E-05
Nme1	0.59	4.90E-05
Plp2	0.47	5.27E-05
Mrpl52	0.97	5.58E-05
F730043M19Rik	1.21	6.25E-05
Zbp1	0.84	6.33E-05
Cdca8	2.57	6.51E-05
Stat1	0.56	6.69E-05
Tgtp1	0.95	7.07E-05
Diablo	0.63	7.11E-05
Rpl32	0.49	7.81E-05
Rpl15-ps3	0.64	7.89E-05
Gm24451	1.05	8.22E-05
Tacc3	2.20	8.34E-05
Rps26	0.46	8.93E-05
Mrpl33	0.49	9.16E-05
Ier2	0.62	9.36E-05
Gm20390	0.68	9.54E-05
Srsf7	0.48	1.03E-04
Cox5a	0.50	1.04E-04
Adpgk	0.77	1.10E-04
Eef1a1	0.44	1.20E-04
Phb	0.58	1.20E-04
Rps17	0.43	1.22E-04
Rps6	0.40	1.25E-04
Atp5md	0.47	1.31E-04
Rpl27	0.43	1.31E-04
Sdf2l1	0.79	1.31E-04
Rpl23a	0.44	1.41E-04
Spc25	3.12	1.46E-04
Cox6c	0.45	1.49E-04
Fam118b	0.66	1.59E-04
Atp5e	0.49	1.61E-04
Txk	0.69	1.71E-04
Eif5a	0.50	1.75E-04
Acap1	0.40	1.78E-04
Snap23	0.71	1.80E-04
Arl5a	0.98	1.86E-04
Racgap1	1.34	1.93E-04
Nlrc5	0.96	1.98E-04
Psme2	0.44	2.07E-04
Tspan31	0.53	2.08E-04
Txn1	0.51	2.08E-04
Rpl3-ps1	0.56	2.10E-04
Gm26917	0.74	2.12E-04
H2afx	0.83	2.22E-04
Sh3bp1	0.46	2.31E-04
Eef2	0.51	2.35E-04
Ecm1	0.69	2.39E-04
S100a4	0.70	2.40E-04
Nek6	1.57	2.50E-04
Ppib	0.42	2.76E-04
D630039A03Rik	0.92	2.86E-04
Klc3	1.16	2.86E-04
Rack1	0.43	2.97E-04
Phgdh	0.75	2.98E-04
Rpl14	0.46	3.01E-04
Rplp0	0.39	3.04E-04
Nhp2	0.63	3.22E-04
Zfp36	0.61	3.39E-04
Hspa8	0.37	3.48E-04

Uhrf1	1.73	3.50E-04
Fen1	0.88	3.57E-04
Mxd3	3.61	3.63E-04
Pcna-ps2	0.79	3.62E-04
Prc1	2.82	3.63E-04
Slc35g1	1.08	3.62E-04
Rpl7a	0.42	3.80E-04
Snhg15	0.58	3.90E-04
Smyd2	0.82	3.95E-04
Mad2l1	1.39	4.11E-04
Hmga1	0.59	4.24E-04
Gm27747	6.31	4.32E-04
Atpif1	0.47	4.52E-04
Rpl23	0.42	4.60E-04
Cox4i1	0.43	4.88E-04
Pglyrp2	0.72	5.60E-04
Fut8	1.17	5.73E-04
Pttg1	1.00	5.79E-04
Sec11c	0.46	5.88E-04
Rps8	0.38	5.94E-04
Bcl3	0.71	5.99E-04
Gm49339	2.28	6.09E-04
Ppia	0.37	6.13E-04
Rpl9	0.37	6.14E-04
Eef1b2	0.37	6.20E-04
Mvb12a	0.57	6.34E-04
Cops9	0.44	6.38E-04
Srgn	0.37	6.81E-04
Csrnp1	0.60	7.12E-04
Cox7a2	0.43	7.41E-04
Selenoh	0.63	7.84E-04
AU020206	0.82	7.93E-04
Gm10499	0.54	8.12E-04
Tespa1	0.72	8.79E-04
Rpl28	0.45	8.86E-04
Gm4332	0.71	9.18E-04
Gm15459	0.37	9.25E-04
Gtse1	2.53	9.31E-04
Pdia3	0.33	9.78E-04
Rps19-ps6	0.36	9.77E-04
Tmem59	0.35	9.79E-04
Gm4739	0.51	9.98E-04
Tpt1	0.37	1.01E-03
Ccng1	0.59	1.03E-03
Anxa1	1.10	1.05E-03
Rpl26	0.35	1.09E-03
Espn	0.75	1.10E-03
Rps6-ps4	0.44	1.11E-03
Anp32b	0.49	1.12E-03
Rps19	0.40	1.12E-03
Rps20	0.46	1.13E-03
Rpl6	0.37	1.20E-03
Rps27a	0.37	1.20E-03
Smc2	1.19	1.22E-03
Mcm3	0.95	1.28E-03
Rpl31	0.42	1.29E-03
Pomp	0.47	1.34E-03
Sla2	0.57	1.36E-03
Tipin	0.66	1.37E-03
Manf	0.47	1.39E-03
Aen	0.67	1.41E-03
Fabp5	0.78	1.44E-03
Gm7666	0.76	1.50E-03
Smdt1	0.46	1.50E-03
Socs3	0.61	1.50E-03
Cks2	0.77	1.55E-03
Capg	0.43	1.57E-03
Gm37881	0.88	1.58E-03
Ighv1-23	4.01	1.62E-03
Itgb3	0.93	1.69E-03
Cd2	0.42	1.76E-03
Prdx4	0.64	1.80E-03
Rtp4	1.77	1.81E-03
Cdca3	2.61	1.84E-03
Tpx2	3.71	1.85E-03
Ccs	0.58	1.92E-03
Pdia6	0.48	1.96E-03
Rpsa	0.35	2.00E-03

Slbp	0.38	2.01E-03
Rps16	0.35	2.04E-03
Rps4x	0.37	2.05E-03
Tnfrsf4	0.43	2.09E-03
Fkbp2	0.62	2.09E-03
1810037I17Rik	0.48	2.11E-03
Bnip3	0.51	2.13E-03
Dcxr	0.87	2.17E-03
Cpe	0.79	2.21E-03
Mdfic	0.87	2.20E-03
Rpl37a	0.36	2.20E-03
Rps7	0.35	2.19E-03
Atp5g3	0.30	2.23E-03
Ifi27l2a	0.63	2.30E-03
Rad51	2.92	2.38E-03
Btf3	0.36	2.49E-03
Ranbp1	0.44	2.48E-03
Tcrg-C1	4.30	2.49E-03
Bst2	0.52	2.50E-03
Nusap1	3.65	2.51E-03
Eef1g	0.40	2.53E-03
Rps3a1	0.31	2.56E-03
Gm11808	0.47	2.58E-03
Gm7125	1.56	2.58E-03
Soat1	0.98	2.58E-03
Commd1	0.61	2.60E-03
Ifi35	0.46	2.63E-03
Lsm4	0.40	2.65E-03
Igfbp7	1.85	2.66E-03
Sypl	0.86	2.70E-03
Gm11223	2.88	2.81E-03
Kif20a	2.50	2.81E-03
Rpl22	0.45	2.83E-03
Fut7	0.93	2.86E-03
Rpl39	0.43	2.87E-03
Agpat3	0.51	2.88E-03
Gm12918	0.36	2.89E-03
Pbk	4.16	2.95E-03
Specc1	1.91	2.97E-03
Sh3bp5	0.57	3.00E-03
Rpl8	0.34	3.01E-03
Tuba1c	0.54	3.04E-03
Aaas	0.64	3.05E-03
Atp5b	0.28	3.05E-03
Rps15	0.33	3.06E-03
Rps10	0.36	3.16E-03
Rpl38	0.40	3.28E-03
Hba-a1	4.81	3.37E-03
Knstrn	2.51	3.38E-03
Ifngr1	0.31	3.46E-03
Rpl35a	0.40	3.52E-03
Gpat4	0.57	3.57E-03
Rpl13a	0.34	3.57E-03
Gm15500	0.38	3.65E-03
Tube1	2.07	3.67E-03
Rpl35	0.40	3.70E-03
Dctpp1	0.78	3.74E-03
Rpl41	0.35	3.80E-03
Ldha	0.33	4.12E-03
Glrx3	0.54	4.14E-03
Plk1	2.95	4.16E-03
Snord13	1.14	4.18E-03
Rfc4	1.35	4.20E-03
Rpl19	0.31	4.31E-03
Rps11	0.34	4.36E-03
Dglucy	0.79	4.43E-03
Gm6472	0.38	4.42E-03
Rasgrp1	0.47	4.41E-03
Rsu1	0.60	4.44E-03
Dbi	0.43	4.49E-03
Gm10123	0.33	4.60E-03
Gm12250	1.10	4.70E-03
Prpf31	0.47	4.83E-03
Cox6a1	0.33	4.86E-03
Lama5	1.63	4.87E-03
Rpl36al	0.33	4.89E-03
H2-D1	0.35	4.90E-03
Anxa6	0.31	4.95E-03

Pgls	0.48	4.99E-03
Kif22	1.06	5.03E-03
Slc25a5	0.33	5.03E-03
Gm10131	0.40	5.16E-03
Mxd1	0.59	5.21E-03
Gm10163	0.47	5.25E-03
Mcm2	0.91	5.27E-03
Rps16-ps2	0.36	5.26E-03
Dkc1	0.41	5.41E-03
H2-T-ps	0.47	5.40E-03
Prr13	0.38	5.48E-03
Gm42418	0.64	5.50E-03
Rps21	0.40	5.53E-03
Nfkb1	0.60	5.58E-03
Ppa1	0.55	5.61E-03
Rplp1	0.35	5.67E-03
Sytl1	0.36	5.67E-03
Mbnl1	0.32	5.72E-03
Hspa5	0.34	5.81E-03
Gm4617	0.54	5.91E-03
Akr1a1	0.32	5.95E-03
Srm	0.46	5.95E-03
Neb	2.01	6.14E-03
Ywhae	0.37	6.15E-03
Wnk1	0.70	6.37E-03
Rps12-ps3	0.96	6.41E-03
Gm10221	0.46	6.47E-03
Tfrc	0.95	6.61E-03
Cnih4	0.43	6.63E-03
Rps2	0.28	6.70E-03
Atp5mpl	0.35	6.96E-03
Gimap3	0.27	6.98E-03
Rpl7	0.30	7.00E-03
Rcan3	0.63	7.02E-03
Sumo3	0.41	7.05E-03
Rpl37	0.39	7.16E-03
Tspan4	2.30	7.16E-03
Ada	1.46	7.28E-03
Rpl13	0.32	7.30E-03
Gm4149	0.44	7.44E-03
Rpl19-ps11	0.36	7.52E-03
Herpud1	0.39	7.72E-03
Sem1	0.43	7.75E-03
Atp5o	0.35	7.87E-03
Pfdn1	0.43	7.86E-03
Gm7536	0.34	7.92E-03
Lig1	1.45	7.98E-03
Gm10260	0.29	8.00E-03
Tomm5	0.34	8.34E-03
Dut	0.66	8.39E-03
Atp5l	0.34	8.51E-03
Prdx1	0.32	8.56E-03
Casp8	0.37	8.58E-03
Ap2s1	0.34	8.64E-03
Dap	0.49	8.64E-03
Fah	1.26	8.60E-03
Gm9794	0.40	8.62E-03
Nek7	0.48	8.76E-03
Sfxn1	0.77	8.78E-03
Gm12715	0.56	8.80E-03
St6galnac2	0.83	8.82E-03
Cdkn2c	1.24	8.87E-03
Smc5	0.67	8.87E-03
Grtp1	1.79	9.03E-03
Gas5	0.27	9.08E-03
Cyba	0.34	9.11E-03
Rhoc	1.08	9.12E-03
Rpl11	0.31	9.19E-03
Hsp90b1	0.41	9.25E-03
Lsm6	0.35	9.29E-03
Cap1	0.39	9.50E-03
H2-T3	0.90	9.73E-03
Fignl1	2.94	9.80E-03

Source data for Fig 5C

EV+IL-2 DE down genes

Gene	Log2FC	PValue
Tcf7	-1.98	8.49E-22
Il7r	-1.44	9.23E-15
Gm8369	-0.85	1.49E-12
St8sia6	-2.12	2.77E-12
Gm10800	-2.77	2.01E-11
1110032F04Rik	-1.70	4.38E-11
Gm21738	-2.73	6.44E-11
Smc4	-0.64	1.96E-10
Cirbp	-0.95	2.70E-10
Otulinl	-0.77	1.11E-09
Tmem191c	-1.06	3.50E-09
Ccr7	-0.70	8.91E-09
Ly9	-0.92	2.05E-08
Rgs2	-1.74	2.38E-08
Rnf167	-0.69	2.71E-08
Sash3	-0.69	4.17E-08
Trbv13-3	-0.69	4.23E-08
Atp1b3	-0.57	8.51E-08
Cd1d1	-0.89	1.28E-07
Cd27	-0.64	1.27E-07
Cd200	-1.19	2.30E-07
Ccr6	-1.33	2.63E-07
Il16	-0.64	2.66E-07
Selenop	-0.88	3.11E-07
Gm10801	-3.22	3.47E-07
Dgka	-0.63	4.20E-07
Itga4	-1.18	4.61E-07
Ypel3	-0.61	6.00E-07
Klhl6	-0.68	9.51E-07
Ets1	-0.57	1.03E-06
Tent5c	-1.95	1.31E-06
Tpr	-0.65	1.36E-06
Btg2	-0.58	2.13E-06
Rflnb	-3.05	2.81E-06
Gm10717	-2.79	3.65E-06
Bcl2a1b	-1.31	4.00E-06
S1pr1	-0.57	4.18E-06
Sat1	-0.46	4.25E-06
Trib2	-0.84	4.26E-06
A630023P12Rik	-0.98	4.68E-06
Ephx1	-1.01	5.04E-06
Crlf3	-0.50	6.02E-06
Tagap	-1.01	6.42E-06
Trbv16	-0.56	6.86E-06
Ehd3	-0.89	7.00E-06
Dnajc7	-0.49	7.54E-06
Arhgap45	-0.54	8.41E-06
Stat5b	-0.76	8.41E-06
Arl5c	-1.06	1.10E-05
Ptp4a2	-0.54	1.21E-05
Jak1	-0.59	1.34E-05
Limd2	-0.52	1.39E-05
Sh3kbp1	-0.64	2.25E-05
B4galnt1	-0.53	2.50E-05
Bmyc	-0.69	2.55E-05
Habp4	-0.86	2.61E-05
Trbv13-1	-0.52	3.08E-05
Tgif1	-0.78	3.13E-05
Smap2	-0.85	3.74E-05
Trbv13-2	-0.50	3.76E-05
Matk	-1.65	3.85E-05
Apol9b	-0.83	7.99E-05
Pdcd1	-0.99	8.41E-05
Cxcr4	-1.76	8.98E-05
Ptpn22	-0.89	9.01E-05
Tsc22d3	-0.61	9.14E-05
Cd28	-0.59	9.42E-05
Dennd2d	-0.63	9.49E-05
Cyth4	-0.43	1.18E-04
Rgs10	-0.65	1.34E-04
Zfp36l1	-0.64	1.38E-04
Trbv15	-0.55	1.41E-04
Ppcdc	-0.62	1.45E-04
9930111J21Rik1	-0.94	1.51E-04
Chchd10	-0.59	1.81E-04
Syt11	-0.92	1.97E-04
Ripor2	-0.55	2.08E-04
Slamf6	-0.66	2.14E-04
Cdk2ap2	-0.51	2.52E-04
Trbv20	-0.64	2.69E-04
Rgs16	-2.41	2.94E-04
Acsbg1	-0.95	3.24E-04
Sigirr	-0.48	3.32E-04
Gimap6	-0.44	3.58E-04
Ighm	-0.69	3.58E-04
Cd96	-0.47	3.81E-04
Adk	-0.64	3.85E-04
Inpp5k	-0.47	4.04E-04
Tk2	-0.65	4.29E-04
Fam3c	-0.72	4.61E-04
Trbc2	-0.42	4.65E-04
Gm45552	-1.10	4.69E-04
Tdrp	-1.39	4.77E-04
Gramd1a	-0.43	5.36E-04
Cd6	-0.42	5.51E-04
Cd52	-0.48	5.69E-04
Akap8l	-0.46	5.84E-04
Ndrg3	-0.62	6.42E-04
Cd5	-0.37	6.46E-04
Gm22243	-5.28	6.73E-04
Fam129a	-0.80	7.10E-04
Cd84	-0.60	7.17E-04
Tnfrsf14	-0.80	8.17E-04
Trbv31	-0.51	8.15E-04
Evl	-0.46	8.61E-04
Sell	-0.39	8.71E-04
Fau-ps2	-2.05	9.48E-04
Trbc1	-0.42	1.04E-03
1600014C10Rik	-0.43	1.06E-03
Sesn1	-0.63	1.08E-03
9930111J21Rik2	-0.83	1.12E-03
Cnn2	-0.37	1.12E-03
Scg5	-1.67	1.15E-03
Trbv4	-0.50	1.16E-03
Pdcd4	-0.46	1.19E-03
Prr14	-0.41	1.24E-03
Lsp1	-0.41	1.25E-03
Sfxn3	-0.49	1.25E-03
Ddx5	-0.41	1.29E-03
Cd9	-0.49	1.37E-03
Pde4b	-0.93	1.38E-03
Tcp11l2	-0.49	1.41E-03
Ppm1b	-0.39	1.48E-03
Mthfsl	-0.52	1.55E-03
Izumo4	-0.57	1.61E-03
Dalrd3	-0.47	1.64E-03
Trbv5	-0.33	1.68E-03
Pea15a	-0.45	1.71E-03
B4galt1	-0.56	1.72E-03
Ift80	-0.55	1.85E-03
Shisa5	-0.45	1.90E-03
Il6ra	-0.50	1.93E-03
Dock2	-0.49	2.14E-03
Gm27718	-5.29	2.21E-03
Trbv2	-0.49	2.26E-03
Akna	-0.40	2.27E-03
Oard1	-0.64	2.28E-03
I730030J21Rik	-0.76	2.39E-03
Pnpla7	-0.62	2.39E-03
Dhrs3	-1.95	2.49E-03
Sla	-0.45	2.59E-03
Pecam1	-0.69	2.64E-03
Bnip3l	-0.36	2.79E-03
Gm10718	-2.70	2.80E-03
Gm11168	-2.76	2.99E-03
Taz	-0.42	3.13E-03
Sntb1	-1.15	3.19E-03
Sirt7	-0.37	3.21E-03
Sqstm1	-0.31	3.33E-03
Traf3ip3	-0.32	3.42E-03
Izumo1r	-0.45	3.81E-03
Trbv17	-0.54	3.81E-03
Zfp869	-0.66	3.96E-03
Arhgef3	-0.58	4.03E-03
Gimap9	-0.37	4.07E-03
Cxcr6	-1.87	4.14E-03
Traf5	-0.54	4.26E-03
Sp100	-0.34	4.34E-03
Stk10	-0.42	4.49E-03
Rsrp1	-0.33	4.52E-03
Orai2	-0.58	4.83E-03
Arhgef1	-0.28	4.86E-03
Gm17173	-0.98	4.92E-03
Txnip	-0.42	4.93E-03
Ltb	-0.41	5.04E-03
Acyp1	-0.74	5.15E-03
Cyth3	-0.92	5.35E-03
Peli1	-0.39	5.39E-03
Ctss	-0.33	5.61E-03
Stk38	-0.38	6.03E-03
Slc28a2	-0.80	6.11E-03
Tgfbr2	-0.70	6.20E-03
Tprgl	-0.34	6.26E-03
Trbv3	-0.44	6.28E-03
Gpcpd1	-0.65	6.30E-03
Mir466j	-5.27	6.34E-03
Mthfs	-0.59	6.43E-03
Serpinb6a	-0.60	6.43E-03
Trex1	-0.54	6.56E-03
Fam189b	-0.36	6.70E-03
Tmc6	-0.54	6.95E-03
Sqor	-0.64	7.43E-03
Fam107b	-0.72	7.61E-03
Spop	-0.42	7.73E-03
Cdc42se2	-0.53	8.13E-03
AI467606	-0.30	8.92E-03
Ptger2	-0.67	8.93E-03
Stat5a	-0.68	9.06E-03
Tmub1	-0.57	9.49E-03
Trbv12-1	-0.58	9.72E-03
Ank	-0.80	9.86E-03
Sdc4	-0.51	9.94E-03

Source data for Fig 5C

3A10t DE up genes

Gene	Log2FC	PValue
Gm10800	2.60	2.39E-10
Gm21738	2.27	1.61E-09
Gm10717	2.70	2.44E-08
Gm10801	3.14	6.00E-07
Ccnd2	0.90	3.00E-06
Socs2	0.46	4.75E-06
Hist1h2ap	0.93	9.55E-06
Gm12191	1.10	1.65E-05
Il2rb	0.49	2.64E-05
Ldha	0.47	3.76E-05
Plec	0.77	4.46E-05
Gm28437	0.48	7.11E-05
Lgals1	0.48	8.68E-05
Tnfrsf4	0.55	8.16E-05
Rpl15-ps3	0.64	1.04E-04
Slc7a6	0.64	1.37E-04
Rexo2	0.48	1.64E-04
Hsd11b1	0.59	1.87E-04
Rplp0	0.39	2.49E-04
Rpl15	0.42	2.63E-04
Ccnd3	0.47	4.13E-04
Gm4735	0.52	4.24E-04
Plp2	0.41	4.17E-04
Hsp90aa1	0.44	4.43E-04
Rpl10	0.39	4.81E-04
Rps5	0.39	4.73E-04
Flt3l	0.55	5.27E-04
Kbtbd11	0.68	5.28E-04
Rpsa	0.39	6.44E-04
Eno1b	0.48	6.89E-04
Actg1	0.35	7.29E-04
Rps6	0.35	8.06E-04
Ndufa4	0.39	8.87E-04
Rpl13a	0.39	8.71E-04
Chchd2	0.40	9.80E-04
Gapdh	0.44	9.96E-04
Gm15427	0.43	9.91E-04
Rps14	0.40	9.58E-04
Rps18	0.37	9.85E-04
Capg	0.44	1.11E-03
Gm15500	0.43	1.11E-03
Ssbp4	0.40	1.07E-03
Atp5o	0.43	1.15E-03
Tespa1	0.71	1.13E-03
Psmd11	0.43	1.22E-03
Tnfrsf18	0.42	1.32E-03
Cox5a	0.41	1.43E-03
Gm8186	0.55	1.43E-03
Rpl23a	0.37	1.42E-03
Rps19-ps6	0.35	1.45E-03
Psmb8	0.46	1.53E-03
Rpl18a	0.41	1.58E-03
Rpl4	0.34	1.57E-03
Akap9	0.59	1.67E-03
Dkc1	0.46	1.86E-03
Igfbp4	1.14	1.90E-03
Ly6e	0.42	1.95E-03
Gm12918	0.37	2.04E-03
Gm37376	0.78	2.22E-03
Myl6	0.37	2.15E-03
Nme1	0.45	2.17E-03
Tnfrsf9	0.55	2.24E-03
Txn2	0.43	2.13E-03
Aprt	0.44	2.35E-03
H2afz	0.36	2.39E-03
Rbm25	0.42	2.39E-03
Rps2	0.32	2.38E-03
Rpl41	0.36	2.41E-03
Atp5l	0.39	2.52E-03
Rps4x	0.36	2.54E-03
Ncf4	0.42	2.64E-03
Prelid1	0.39	2.63E-03
Ndufa10	0.43	2.88E-03
Gm9794	0.46	3.08E-03
Crip1	0.38	3.15E-03
Rps11	0.36	3.15E-03
Eno1	0.40	3.27E-03
Arpc1b	0.38	3.34E-03
Cd3g	0.34	3.48E-03
Rpl10a	0.35	3.37E-03
S100a4	0.56	3.40E-03
Slc25a5	0.34	3.49E-03
Tpi1	0.45	3.46E-03
Glipr1	0.40	3.72E-03
Ptpn1	0.43	3.74E-03
Rps9	0.32	3.68E-03
Stmn1	0.88	3.67E-03
Uqcrh	0.33	3.74E-03
Rab14	0.38	3.80E-03
S100a6	0.38	3.82E-03
Rpl13	0.35	3.87E-03
Cd2	0.39	4.07E-03
Gm10131	0.42	3.99E-03
Rpl14	0.37	4.00E-03
Srgn	0.32	4.02E-03
Tagln2	0.36	4.18E-03
Oaz1-ps	0.40	4.21E-03
Uqcrb	0.34	4.24E-03
Dennd1c	0.43	4.29E-03
Rps27l	0.36	4.37E-03
Ndufb9	0.36	4.49E-03
Psme1	0.36	4.46E-03
Gm13394	0.54	4.57E-03
Rps17	0.32	4.56E-03
Ndufb8	0.38	4.68E-03
Swi5	0.39	4.64E-03
mt-Nd3	0.76	4.67E-03
Il2rg	0.34	4.82E-03
Pepd	0.49	4.80E-03
Snx3	0.35	4.84E-03
Ifngr1	0.30	4.98E-03
Rps3a1	0.29	5.02E-03
Cox4i1	0.35	5.12E-03
Ptma	0.29	5.16E-03
Emp3	0.37	5.20E-03
Gm10039	0.47	5.24E-03
Dok2	0.36	5.33E-03
Trac	0.35	5.32E-03
Rpl3-ps1	0.43	5.44E-03
Sit1	0.41	5.50E-03
Atp5md	0.34	5.55E-03
Cops9	0.36	5.58E-03
Gm2000	0.38	5.61E-03
Cdkn2d	0.41	6.03E-03
Ifitm3	0.82	6.03E-03
Ndufa5	0.44	6.04E-03
Cd52	0.38	6.30E-03
Rpl32	0.34	6.53E-03
Cox6a1	0.32	6.59E-03
Grap2	0.45	6.87E-03
Rpl26	0.29	6.85E-03
Rps15	0.30	6.96E-03
Ndufv3	0.37	7.01E-03
Malat1	0.78	7.05E-03
Izumo1r	0.40	7.17E-03
Srrm2	0.54	7.19E-03
Rnf125	0.44	7.44E-03
Grap	0.36	7.53E-03
Cox6c	0.32	7.71E-03
Gm10260	0.29	7.66E-03
Batf	0.42	7.81E-03
mt-Tm	1.86	7.78E-03
Eif3k	0.37	8.08E-03
Lta	0.38	8.02E-03
Mif	0.32	7.97E-03
Rpl35	0.37	7.94E-03
Rps6-ps4	0.36	8.18E-03
Commd4	0.37	8.29E-03
Pop5	0.34	8.41E-03
Stk16	0.38	8.42E-03
Ndufb7	0.36	8.48E-03
Prdx6	0.32	8.47E-03
Atp5c1	0.31	8.69E-03
Cox7a2	0.34	8.66E-03
Dynlrb1	0.35	8.78E-03
Gm10163	0.44	8.72E-03
Gm10288	0.40	8.82E-03
Rab11a	0.36	8.58E-03
Rps23-ps1	0.34	8.79E-03
Tmed9	0.37	8.91E-03
Gm11808	0.41	9.34E-03
Fam162a	0.36	9.41E-03
Drc1	0.53	9.61E-03
Lsm7	0.38	9.66E-03
Tpt1	0.29	9.72E-03
Cope	0.35	1.00E-02

Source data for Fig 5C

3A10t DE down genes

Gene	Log2FC	PValue
Thy1	*-3.83*	*4.26E-191*
**Tcf7**	-1.02	7.42E-08
**Rbl2**	-0.91	2.61E-07
**Stk10**	-0.73	1.64E-06
**Wac**	-1.06	2.14E-06
**Ptprc**	-0.57	4.18E-06
**Ift80**	-0.80	1.04E-05
**Ppp2r5c**	-0.69	1.11E-05
**Atp2a3**	-0.75	1.39E-05
**Matr3**	-0.64	1.66E-05
**Eif4g2**	-0.80	2.48E-05
**9930111J21Rik1**	-1.05	2.98E-05
**Ripor2**	-0.64	2.85E-05
**Stat6**	-0.69	3.02E-05
**Ddb1**	-0.71	3.91E-05
**Dock2**	-0.64	7.82E-05
**Acly**	-0.76	8.56E-05
**Tnfrsf1b**	-0.75	1.32E-04
**Rasal3**	-0.48	1.96E-04
**Arhgef3**	-0.77	2.22E-04
**Clint1**	-0.73	2.75E-04
**Pank4**	-0.75	3.29E-04
**Tcp11l2**	-0.55	3.88E-04
**Psd4**	-0.74	4.32E-04
**Api5**	-0.71	4.86E-04
**Efr3a**	-0.61	4.72E-04
**Pip5k1c**	-0.72	5.69E-04
**Edc4**	-0.78	6.60E-04
**Add1**	-0.53	7.28E-04
**Ikbke**	-0.55	8.13E-04
**Copb1**	-0.70	8.42E-04
**Xpo6**	-0.69	8.34E-04
**Csde1**	-0.64	9.08E-04
**Nckap1l**	-0.75	9.10E-04
**Slc38a2**	-0.71	9.01E-04
**Atp11b**	-0.69	9.46E-04
**Caprin1**	-0.58	1.09E-03
**Usp19**	-0.65	1.11E-03
**Flii**	-0.67	1.20E-03
**Stk24**	-0.64	1.22E-03
**Hif1a**	-0.53	1.25E-03
**Nt5e**	-0.67	1.29E-03
**Copa**	-0.62	1.37E-03
**Txnip**	-0.47	1.40E-03
**Actr2**	-0.50	1.55E-03
**Ccng1**	-0.62	1.56E-03
**Rpl30-ps9**	-0.43	1.73E-03
**Mfsd14a**	-0.60	1.89E-03
**Ldb1**	-0.80	1.98E-03
**Sidt2**	-0.59	1.97E-03
**Arcn1**	-0.55	2.04E-03
**Dnajc7**	-0.33	2.09E-03
**Ip6k1**	-0.61	2.16E-03
**Map4k2**	-0.54	2.23E-03
**Rnf14**	-0.64	2.15E-03
**Tmem30a**	-0.63	2.07E-03
**Tpp2**	-0.63	2.19E-03
**Adamts10**	-0.51	2.39E-03
**Cab39**	-1.05	2.28E-03
**Ikzf1**	-0.54	2.32E-03
**Sla**	-0.46	2.29E-03
**Znrf1**	-0.50	2.47E-03
**Arhgap45**	-0.36	2.57E-03
**Dgkz**	-1.02	2.56E-03
**Tnfrsf26**	-0.71	2.67E-03
**Flna**	-0.50	2.74E-03
**Atp13a1**	-0.49	2.88E-03
**Cdc42se2**	-0.60	2.85E-03
**Elf1**	-0.55	2.89E-03
**Fam102a**	-0.55	2.82E-03
**Serinc3**	-0.60	3.17E-03
**Ccng2**	-0.54	3.25E-03
**Ablim1**	-0.42	3.50E-03
**Rasa1**	-0.58	3.52E-03
**Tsc22d4**	-0.41	3.49E-03
**Sept9**	-0.38	3.54E-03
**Tgoln1**	-0.61	3.60E-03
**Irak1**	-0.59	4.06E-03
**Pdcd4**	-0.41	3.97E-03
**Sash3**	-0.36	3.94E-03
**Cpsf1**	-0.58	4.34E-03
**Lbr**	-0.52	4.41E-03
**P4ha1**	-0.56	4.47E-03
**Pdcd6ip**	-0.50	4.51E-03
**Arhgef1**	-0.28	4.73E-03
**Ost4**	-0.30	4.71E-03
**Ptp4a1**	-0.66	4.76E-03
**Sec24c**	-0.48	4.76E-03
**Trim59**	-0.65	5.08E-03
**Ptpra**	-0.57	5.14E-03
**Pik3cd**	-0.45	5.21E-03
**Arhgap4**	-0.48	5.43E-03
**Tmem71**	-0.61	5.50E-03
**Ubqln1**	-0.59	5.42E-03
**Usp4**	-0.52	5.47E-03
**Polg**	-0.61	5.61E-03
**Lrrc8c**	-0.49	5.67E-03
**Amfr**	-1.01	6.00E-03
**Gak**	-0.58	5.96E-03
**Trpc4ap**	-0.65	6.08E-03
**Strn4**	-0.50	6.17E-03
**Tagap1**	-0.61	6.18E-03
**Cbx3**	-0.45	6.68E-03
**Mettl17**	-0.45	6.67E-03
**Qrich1**	-0.48	6.79E-03
**Cyth1**	-0.38	6.93E-03
**Add3**	-0.39	7.08E-03
**Hnrnpu**	-0.39	7.21E-03
**Il4ra**	-0.44	7.14E-03
**Pnpla7**	-0.55	7.41E-03
**Eif4a2**	-0.58	7.48E-03
**Washc2**	-0.52	7.59E-03
**Cul1**	-0.54	7.73E-03
**Nek7**	-0.54	7.68E-03
**Ehd3**	-0.51	8.07E-03
**Sf3b3**	-0.46	7.93E-03
**Slc44a2**	-0.33	8.07E-03
**Ss18**	-0.43	7.92E-03
**Rasa3**	-0.66	8.75E-03
**Srsf1**	-0.49	8.75E-03
**Nxf1**	-0.39	9.35E-03
**Arhgap30**	-0.48	9.47E-03
**Ly9**	-0.41	9.53E-03
**Ddx5**	-0.33	9.64E-03
**S1pr1**	-0.32	9.57E-03
**Shc1**	-0.53	9.77E-03

Genes listed in italicized font are out of the scale in Fig. 5C

Source data for Fig 5C

1G12t DE up genes

Gene	Log2FCh	PValue
Gm22117	*22.04*	*2.12E-07*
**Il2rb**	0.58	6.15E-07
**Il2**	6.69	1.54E-06
**Fos**	3.00	1.02E-03
**Gm10243**	1.42	5.56E-04
Gm24105	*7.18*	*5.73E-04*
**Gm27747**	5.75	1.38E-03
**Tnfrsf9**	0.46	9.47E-03

Genes listed in italicized font are out of the scale in Fig. 5C

Source data for Fig 5C

1G12t DE down genes

Gene	Log2FC	PValue
Thy1	*-3.96*	*1.03E-205*
Traj40	*-21.33*	*5.16E-07*
**Crlf3**	-0.32	3.51E-03
**Gm27718**	-5.37	1.90E-03
**Gm28661**	-0.40	8.04E-03
**Gm47441**	-0.88	2.69E-03
**Rpl30-ps9**	-0.44	1.05E-03
**Sell**	-0.32	7.02E-03
Snord35a	*-7.34*	*2.13E-04*
Trav7d-2	*-7.24*	*1.49E-03*
**Ywhaq-ps3**	-1.48	1.55E-03

Genes listed in italicized font are out of the scale in Fig. 5C
