# Supplementary material for: Receptor-tethered orthogonal IL-2 enhances regulatory T cell therapy
Source: J Exp Med. 2026 Aug 12;223(9):e20251480. doi: 10.1084/jem.20251480 (PMC13521516; doi:10.1084/jem.20251480)
Supplement: Supplementary file 2 [file jem_20251480_sourcedataf7.pdf]

Source data for Fig. 7c

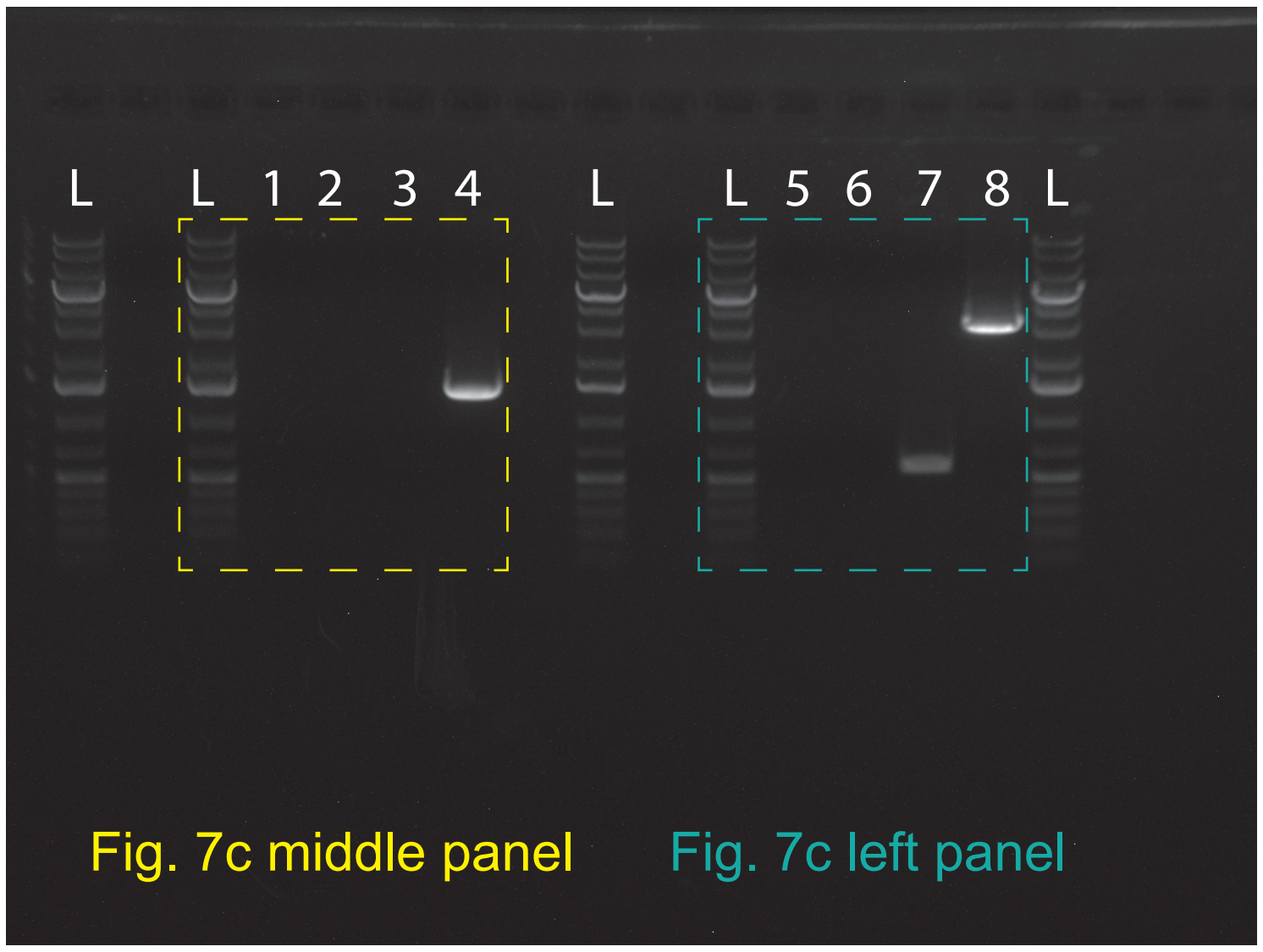

L: DNA ladder
blank
L: DNA ladder
1: Tregs, no RNP or AAV5, Exon 13 to 3A10t PCR
2: Tregs, RNP only, Exon 13 to 3A10t PCR
3: Tregs, RNP+AAV5 vector, Exon 13 to 3A10t PCR
4: Tregs, RNP+AAV5-3A10t, Exon 13 to 3A10t PCR
blank
L: DNA ladder
blank
L: DNA ladder
5: Tregs, no RNP or AAV5, Exon 13 to Thy1.1 PCR
2: Tregs, RNP only, Exon 13 to Thy1.1 PCR
3: Tregs, RNP+AAV5 vector, Exon 13 to Thy1.1 PCR
4: Tregs, RNP+AAV5-3A10t, Exon 13 to Thy1.1 PCR
L: DNA ladder

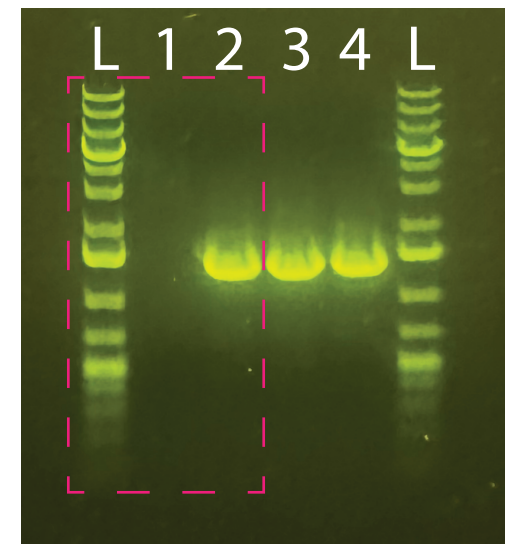

Fig. 7c right panel

L: DNA ladder
1: Tconvs, no RNP or AAV5, Exon 13 to 3A10t PCR
2: Tconvs, RNP+AAV5-3A10t, Exon 13 to 3A10t PCR
*3: iTregs, RNP+AAV5-3A10t, Exon 13 to 3A10t PCR
*4: iTregs, RNP+AAV5-3A10t, Exon 13 to 3A10t PCR
L: DNA ladder
* these data were not part of this manuscript
