# Supplementary material for: Receptor-tethered orthogonal IL-2 enhances regulatory T cell therapy
Source: J Exp Med. 2026 Aug 12;223(9):e20251480. doi: 10.1084/jem.20251480 (PMC13521516; doi:10.1084/jem.20251480)
Supplement: Supplementary file 3 [file jem_20251480_tables1.docx]

**Table S1. Key cell culture reagents.**

| **Reagents** | **Company** | **Catalog number** |
| --- | --- | --- |
| PlatE cells | Cell-Biolabs | RV101 |
| Puromycin | Sigma Aldrich | P8833 |
| Blasticidin | Thermo Fisher | A11139-03 |
| Polybrene | Sigma Aldrich | TR-1003-G |
| Lipofectamine 2000 | Thermo Fisher | 11668027 |
| Opti-MEM | Thermo Fisher | 31985062 |
| Easy sep mouse CD4+ Tcell isolation kit | Stem Cell Technologies | 19852A |
| Pan T cell isolation kit II, mouse | Miltenyi Biotec | 130-095-130 |
| Anti-CD3 (145-2C11) | Thermo Fisher | 14003182 |
| Anti-CD28 (PV-1) | In house | N/A |
| Dynabeads Mouse T-Activator CD3/CD28 for T-Cell Expansion and Activation | Thermo Fisher | 11452D |
| rhIL2 (Proleukin) | Chiron Therapeutics | N/A |
| Sodium pyruvate (100mM) 100X | Gibco | 11360070 |
| MEM NEAA (100X) | Gibco | 11140-050 |
| HEPES (1M) | Gibco | 15630080 |
| FBS | Omega Scientific | FB-01 |
| DMEM | Gibco | 11960-044 |
| Pen strep | Gibco | 15140-122 |
