# Supplementary material for: Receptor-tethered orthogonal IL-2 enhances regulatory T cell therapy
Source: J Exp Med. 2026 Aug 12;223(9):e20251480. doi: 10.1084/jem.20251480 (PMC13521516; doi:10.1084/jem.20251480)
Supplement: Supplementary file 4 [file jem_20251480_tables2.docx]

**Table S2. Key molecular biology reagents.**

| **Reagents** | **Company** | **Catalog number** |
| --- | --- | --- |
| Retro-X | Takara | 631455 |
| Buffer TCL, 2X | Qiagen | 1070498 |
| VAHTS RNA Clean Beads | Vazyme | N412 |
| Discover-sc WTA kit V2 | Vazyme | N711 |
| VAHTS DNA Clean Beads | Vazyme | N411 |
| TruePrep DNA Library Prep Kit V2 for Illumina | Vazyme | TD501 |
| TruePrep Index Kit V2 for Illumina | Vazyme | TD202 |
| Benzonase | Millipore Sigma | 70-664-3 |
| OptiPrep™ (Iodixanol) Density Gradient Medium | StemCell Technologies | 7820 |
| Amicon® Ultra-15 Centrifugal Filter Unit | Amicon | UFC901024 |
| Cas9-NLS purified protein | QB3 Macrolab | N/A |
| P3 Primary Cell 96-well Nucleofector® Kit | Lonza | V4SP-3960 |
| Phusion high Fidelity DNA polymerase | NEB | M0530S |
| T4 DNA ligase | NEB | M0202S |
| NEBuilder HIFI DNA assembly cloning kit | NEB | E5520 |
| Zymo pure ii plasmid maxiprep kit | Zymo | D4203 |
| LB agar plates with carbenicillin-100 | Teknova | L1010 |
| QIA prep spin miniprep kit | Qiagen | 27104 |
| QIA quick extraction kit | Qiagen | 28704 |
| MyTaq™ HS Red Mix | Meridian Bioscience | BIO-25048 |
