# Supplementary material for: Receptor-tethered orthogonal IL-2 enhances regulatory T cell therapy
Source: J Exp Med. 2026 Aug 12;223(9):e20251480. doi: 10.1084/jem.20251480 (PMC13521516; doi:10.1084/jem.20251480)
Supplement: Supplementary file 5 [file jem_20251480_tables3.docx]

**Table S3. Amino acid sequences of various IL-2 and IL-2R constructs.**

| **Constructs** | **Amino Acid Sequences** |
| --- | --- |
| Ortho-IL-2b | AVKNCSHLECFYNSRANVSCMWSHEEALNVTTCHVHAKSNLRHWNKTCELTLVRQASWACNLILGSFPESQSLTSVDLLDINVVCWEEKGWRRVKTCDFHPFDNLRLVAPHSLQVLHIDTQRCNISWKVSQVSDFIEPYLEFEARRRLLGHSWEDASVLSLKQRQQWLFLEMLIPSTSYEVQVRVKAQRNNTGTWSPWSQPLTFRTRPADPMKEILPMSWLRYLLLVLGCFSGFFSCVYILVKCRYLGPWLKTVLKCHIPDPSEFFSQLSSQHGGDLQKWLSSPVPLSFFSPSGPAPEISPLEVLDGDSKAVQLLLLQKDSAPLPSPSGHSQASCFTNQGYFFFHLPNALEIESCQVYFTYDPCVEEEVEEDGSRLPEGSPHPPLLPLAGEQDDYCAFPPRDDLLLFSPSLSTPNTAYGGSRAPEERSPLSLHEGLPSLASRDLMGLQRPLERMPEGDGEGLSANSSGEQASVPEGNLHGQDQDRGQGPILTLNTDAYLSLQELQAQDSVHLI |
| Ortho-IL-2 3A10 | APTSSSTSSSTAEAQQQQQQQQQQQQHLDNLLVLLKALLSRMENYRNLKLPRMLTFKFYLPKQATELKDLQCLEDELGPLRHVLDLTQSKSFQLEDAENFISNIRVTVVKLKGSDNTFECQFDDESATVVDFLRRWIAFCQSIISTSPQ |
| Ortho-IL-2 1G12 | APTSSSTSSSTAEAQQQQQQQQQQQQHLENLLVLLTHLLSKMENYRNLKLPRMLTFKFYLPKQATELKDLQCLEDELGPLRHVLDLTQSKSFQLEDAENFISNIRVTVVKLKGSDNTFECQFDDESATVVDFLRRWIAFCQSIISTSPQ |
| wt-IL-2Rb | AVKNCSHLECFYNSRANVSCMWSHEEALNVTTCHVHAKSNLRHWNKTCELTLVRQASWACNLILGSFPESQSLTSVDLLDINVVCWEEKGWRRVKTCDFHPFDNLRLVAPHSLQVLHIDTQRCNISWKVSQVSHYIEPYLEFEARRRLLGHSWEDASVLSLKQRQQWLFLEMLIPSTSYEVQVRVKAQRNNTGTWSPWSQPLTFRTRPADPMKEILPMSWLRYLLLVLGCFSGFFSCVYILVKCRYLGPWLKTVLKCHIPDPSEFFSQLSSQHGGDLQKWLSSPVPLSFFSPSGPAPEISPLEVLDGDSKAVQLLLLQKDSAPLPSPSGHSQASCFTNQGYFFFHLPNALEIESCQVYFTYDPCVEEEVEEDGSRLPEGSPHPPLLPLAGEQDDYCAFPPRDDLLLFSPSLSTPNTAYGGSRAPEERSPLSLHEGLPSLASRDLMGLQRPLERMPEGDGEGLSANSSGEQASVPEGNLHGQDQDRGQGPILTLNTDAYLSLQELQAQDSVHLI |
| wt-IL2 | APTSSSTSSSTAEAQQQQQQQQQQQQHLEQLLMDLQELLSRMENYRNLKLPRMLTFKFYLPKQATELKDLQCLEDELGPLRHVLDLTQSKSFQLEDAENFISNIRVTVVKLKGSDNTFECQFDDESATVVDFLRRWIAFCQSIISTSPQ |
| CD25r linker | EREHHRFLASEESQGSRNSSPESETSC |
| CD25f linker | SQGSRNSSPESETSCPITTTDFPQP |
| GS3 linker | GGGGSGGGGSGGGGS |
| GS4 linker | GGGGSGGGGSGGGGSGGGGS |
