# Supplementary material for: Receptor-tethered orthogonal IL-2 enhances regulatory T cell therapy
Source: J Exp Med. 2026 Aug 12;223(9):e20251480. doi: 10.1084/jem.20251480 (PMC13521516; doi:10.1084/jem.20251480)
Supplement: Supplementary file 6 [file jem_20251480_tables4.docx]

**Table S4. Flow cytometry reagents.**

| **Marker** | **Fluorophore** | **Clone** | **Company** | **Catalog Number** |
| --- | --- | --- | --- | --- |
| CD25 | BUV395 | PC61 | BD Biosciences | 564022 |
| CD25 | PE-Cyanine7 | PC61.5 | Thermo Fisher Scientific | 25025182 |
| CD122 | e450 | TM-b1 | Thermo Fisher Scientific | 48122282 |
| CD3 | BUV737 | 17A2 | BD Biosciences | 612803 |
| CD4 | APC | GK1.5 | Biolegend | 100412 |
| CD4 | AF780 | RM4-5 | Thermo Fisher Scientific | 47-0042-82 |
| CD4 | FITC | GK1.5 | Thermo Fisher Scientific | 11-0041-82 |
| CD4 | BUV737 | RM4-5 | BD Biosciences | 612844 |
| CD4 | BV605 | RM4-5 | Biolegend | 100547 |
| CD44 | PE | IM7 | BD Biosciences | 553134 |
| CD44 | PerCP-Cyanine5.5 | IM7 | Thermo Fisher Scientific | 450044182 |
| CD45 | PE-Cyanine7 | 30-F11 | Biolegend | 103114 |
| CD45 | BUV395 | 30-F11 | BD Biosciences | 564279 |
| CD62L | AF647 | MEL-14 | Biolegend | 104421 |
| CD8a | e450 | 53-6.7 | Thermo Fisher Scientific | 48008182 |
| CD8a | PerCP-Cyanine5.5 | 53-6.7 | Thermo Fisher Scientific | 45008182 |
| CD8 | PERCP | 53-6.7 | Biolegend | 100732 |
| BCL2 | PE-Cyanine7 | 10-C4 | Thermo Fisher Scientific | 25699242 |
| CTLA4 | PE | UC10-4F10-11 | BD Biosciences | 553720 |
| FOXP3 | AF488 | FJK-16S | Thermo Fisher Scientific | 53577382 |
| THY1.1 | PACIFIC BLUE | OX-7 | Biolegend | 202522 |
| THY1.1 | FITC | OX-7 | Biolegend | 202503 |
| THY1.1 | BV605 | OX-7 | Biolegend | 202537 |
| THY1.1 | APC | OX-7 | Biolegend | 202526 |
| THY1.2 | PE | 53-2.1 | Thermo Fisher Scientific | 12090283 |
| pSTAT5 | AF647 | pY694 | BD Biosciences | 612599 |
| pAKT | PE | Clone J1-223.371 | BD Biosciences | 558275 |
| pSTAT3 | AF488 | 13A3-1 | Biolegend | 651006 |
| pSTAT6 | PE | Clone J71-773.58.11 | BD Biosciences | 558252 |
| True-Phos™ Perm Buffer | | | Biolegend | 425401 |
| eBioscience Foxp3 transcription factor staining buffer set | | | Thermo Fisher | 552300 |
| eBioscience™ Cell Proliferation Dye eFluor™ 450 | | | Invitrogen | 65-0842-85 |
